# Residual Whole-Body Control for Contact-Aware Humanoid Loco-Manipulation with Phase-Conditioned Motion Priors

**DOI:** 10.3390/s26216769

**Published:** 2026-10-26

**Authors:** Han Zhou, Lambert Luo, Edward H. Zhang

**Affiliations:** 1 Faculty of Architecture, Building and Planning, The University of Melbourne, Melbourne, VIC 3053, Australia; hanzhou3@student.unimelb.edu.au; 2 Department of Physics, University of California, Berkeley, Berkeley, CA 94720, USA; 3 Department of Mechanical Engineering, Carnegie Mellon University, Pittsburgh, PA 15213, USA

**Keywords:** humanoid loco-manipulation, residual learning, motion priors, object-state observations, hazard-weighted reinforcement learning

## Abstract

Humanoid object transport couples high-dimensional locomotion with non-smooth contact interactions. Retargeted demonstration priors can fail under contact mismatches, payload changes, and dynamic perturbations. We present residual learning for humanoid object carrying (ResCarry), a residual whole-body control framework built on phase-conditioned motion priors. It first trains an object-centric command prior with vector-quantized codebooks and a causal transformer to capture multi-phase interactions. The prior is then fixed, and a bounded residual whole-body policy learns joint-reference corrections from proprioceptive, contact, and object-state feedback. To reduce catastrophic failures, an empirical upper-tail hazard metric from a multi-atom quantile critic adapts the expected-cost penalty in proximal policy optimization (PPO). A deployable risk estimator slows command progression in high-hazard states. Across six contact-specified furniture manipulation tasks, ResCarry achieves 59.7% success in Isaac Lab. Zero-shot transfer to Unitree MuJoCo reaches 54.8%.

## 1. Introduction

Humanoid robots in human-centered environments must coordinate mobility and manipulation, not treat them separately. Object carrying is a representative whole-body task: the robot approaches an object, establishes contact, moves the load, and places it without losing balance. External end-effector loads alter locomotion and couple manipulation to balance regulation [1]. Interaction-aware whole-body control has likewise been studied for compliant object transport [2]. Unified loco-manipulation control requires coordinated upper- and lower-body responses [3]. Bulky objects may demand simultaneous use of the hands, arms, and torso [4]. Carrying is more than walking plus arm tracking.

The floating base, intermittent foot contacts, upper-body motion, and manipulated object are tightly coupled. Small wrist pose errors can prevent a bilateral hold from seating; torso oscillation can cause one-sided contact loss or slip; errors accumulated across footsteps can end in a drop. Payload mass and inertia, friction, grasp geometry, command delay, and walking direction also change the correction needed around the same nominal motion. A controller must maintain feasible whole-body motion and stable object contact despite falls, grasp losses, collisions, and drops. Good average tracking does not prevent these failures. Human–object demonstrations reduce exploration in this high-dimensional setting. HDMI (a human-video-based whole-body interaction learning system) and HuMI (Humanoid Manipulation Interface) show both the value of whole-body interaction demonstrations and the scarcity of contact-rich humanoid–object data [5,6]. HumanoidMimicGen is a demonstration-generation framework that expands limited source demonstrations through planning in the composite leg–torso–arm action space [7]. In this work, OMOMO (Object Motion guided human Motion synthesis) provides human and object trajectories [8], and interaction-preserving retargeting converts them into robot-compatible references [9]. The resulting data retain phase structure and object-relative coordination that reinforcement learning (RL) would otherwise have to learn from scratch.

A motion prior alone is not enough for reliable carrying. Human-to-robot retargeting cannot exactly preserve contact geometry, actuator limits, balance margins, impact timing, or the dynamics of a particular robot–object pair. ResMimic (residual mimicry for humanoid loco-manipulation) reports that general motion tracking lacks the precision and object awareness needed for loco-manipulation, motivating residual interaction refinement [10]. SEEC (stable end-effector control) similarly shows how bipedal locomotion disturbances can degrade end-effector accuracy even with a well-defined nominal arm motion [11]. Residual reinforcement learning fits this two-part design: a nominal controller or learned prior provides structured behavior, and learning supplies corrections for contact, friction, model error, and interaction dynamics [12]. For carrying, the prior retains its phase structure and a bounded corrective policy handles object interaction and balance recovery. Average return also misses rare drops and falls. Formal risk-sensitive policy optimization addresses tail outcomes through objectives such as conditional value-at-risk (CVaR) [13]. Our method does not optimize a direct CVaR policy gradient or claim convergence as a constrained Markov decision process (CMDP) solver. A quantile critic represents the distribution of discounted hazard cost. Its upper-tail average provides an empirical CVaR estimate for a Lagrangian-style update of the expected-cost proximal policy optimization (PPO) coefficient. A lightweight estimator uses deployable observations to slow or pause nominal-command progression when the estimated hazard is high.

We present ResCarry, a two-stage residual learning framework for contact-aware humanoid object carrying. Stage I learns a phase-conditioned object-centric command prior from retargeted human–object motions and teacher-augmented trajectories. It generates base, torso, wrist, and gripper commands, then remains fixed during reinforcement learning training. Stage II converts each command into a nominal whole-body reference and learns bounded joint-reference corrections from proprioceptive, contact, and object-relative feedback. Fixing the prior separates reference generation from contact-conditioned execution. The residual policy then accounts for interaction errors, payload variation, and whole-body dynamics. The current implementation uses the 29 degrees of freedom (DoF) Unitree G1; we do not claim cross-platform hardware generalization.

We evaluate six pose/identifier-specified furniture interaction and carrying tasks. Object pose and twist come from a simulator interface with randomized noise and latency; image perception and object-state estimation are not evaluated. We compare the fixed command prior, a coarse-reference residual controller, continued supervised training of the command prior, and a fixed expected-cost residual baseline. Component removals, payload tests, beyond-randomization estimator diagnostics, and zero-update transfer from Isaac Lab to Unitree MuJoCo probe different parts of the implementation. The study examines whether fixed motion priors, contact-aware residual correction, and empirical upper-tail hazard weighting work well together for simulated humanoid carrying. It does not aim to establish general-purpose humanoid manipulation. The main contributions are:

- The controller is organized as a two-stage whole-body carrying formulation: it preserves a phase-conditioned object-centric command prior and learns bounded residual corrections from proprioceptive, contact, and object-state observations.
- Hazard handling uses a practical PPO implementation in which a quantile hazard critic supplies an upper-tail score for adapting the expected-cost penalty; a deployable hazard score estimator additionally regulates command progression.
- Evidence is provided through a controlled simulation evaluation on six contact-specified furniture tasks, including matched reference and training variants, component-removal studies, payload tests, and zero-update Isaac Lab–to–MuJoCo evaluation under a fixed observation interface.

## 2. Related Work

### 2.1. Humanoid Loco-Manipulation

Recent work has improved humanoid loco-manipulation through end-to-end learning, force-aware control, and demonstration-based control. Early sim-to-real results demonstrated that box pickup and transport can be learned end-to-end and transferred to hardware, although they relied on carefully engineered task structure and reward shaping [14]. FALCON (force-adaptive loco-manipulation control) decomposes upper- and lower-body control to adapt to measured or inferred end-effector interaction forces, improving robustness to unmodeled payload dynamics [1]. Other work combines model-based task generation with learned whole-body tracking for robust execution [15]. TRILL is a teleoperation-based humanoid loco-manipulation learning framework that collects demonstrations and stabilizes high-level commands with whole-body control [16]. In human–robot collaboration, HALO denotes heterogeneous-agent Lyapunov policy optimization and studies coordinated safety-aware learning [17].

### 2.2. Motion Priors and Residual Control

Motion priors reduce the amount of task-specific exploration needed for contact-rich whole-body skills. Recent frameworks use single-demonstration transfer and whole-body motion planning to expand training data [7,18]. Diffusion-guided augmentation and reinforcement learning fine-tuning have also been applied to generative action models [19,20]. Transformer and diffusion action models can serve as short-horizon controllers or motion priors in manipulation [21,22]. Transfer learning has also been used to adapt energy-management policies across operating conditions in electric vehicles [23]. Our controlled variants use the same Stage I phase imitation-learning (IL) prior, so the comparisons isolate execution-side changes.

ResCarry fixes this phase-conditioned command prior and trains a residual policy with explicit hazard costs for fall, drop, grasp loss, collision, slip, and joint-limit events. The actor observes joint, inertial measurement unit (IMU), contact, and object-state quantities. Training uses the official Unitree G1 assets in Isaac Lab [24,25], and the unchanged policy is evaluated in the official Unitree MuJoCo environment [26,27].

### 2.3. Risk-Sensitive Reinforcement Learning

Model-free RL for legged and humanoid locomotion typically uses PPO for its stable clipped policy updates [28], and most humanoid loco-manipulation work inherits its expected-return objective. Multi-scale RL has also been applied to dynamic energy control in connected electrified vehicles [29]. Tail outcomes matter for reliability. Constrained policy optimization introduces an explicit expected-cost constraint and a first-order feasible update direction [30]. Risk-sensitive RL more directly targets tail behavior through CVaR objectives, which provide convex, coherent risk measures for Lagrangian optimization [31]. Distributional RL estimates the return distribution, not just its mean, giving access to quantiles, variance, and tail statistics that expected-value methods discard [32]. Quantile regression offers a practical non-parametric fit with a fixed number of quantile atoms and a pinball/Huber regression loss [33].

Our implementation uses a multi-atom quantile critic [33] for discounted hazard cost. The upper-tail mean updates a nonnegative penalty coefficient. The actor still uses clipped reward- and expected-cost-advantage terms. The quantile tail average is the standard empirical CVaR estimator of discounted hazard cost and drives a projected dual-ascent update. PPO with neural networks is non-convex, and the primal step uses a clipped expected-cost surrogate, not an exact CVaR gradient. We therefore refer to the resulting approximate Lagrangian schedule as hazard-weighted RL; it does not claim strong duality or CMDP convergence. A deployable estimator approximates the normalized hazard score from the actor observation and adjusts command progression.

## 3. Methodology

**Figure 1:**
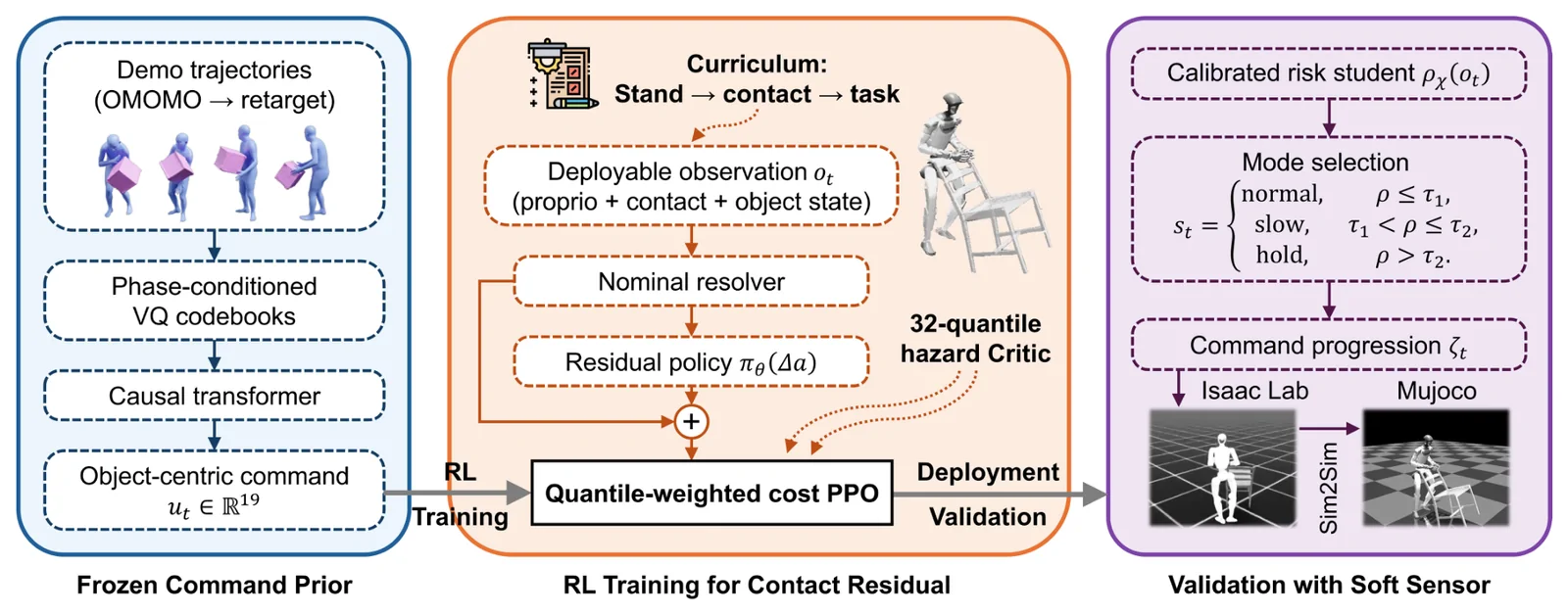
Overview of ResCarry. Stage I: Phase-conditioned command prior. Stage II: Residual whole-body policy, quantile hazard critic (training only), deployable hazard score estimator, and adaptive command progression. Training in Isaac Lab is followed by zero-update cross-engine evaluation in Unitree MuJoCo.

ResCarry is trained in two stages. Stage I (Section 3.3) yields a fixed phase-conditioned command prior $\pi_{\mathrm{pri}}$ that produces $u_{t}$. Stage II (Section 3.4 and Section 3.5) trains a residual tracker $\pi_{\theta }$ with a quantile-based hazard-weighting rule. Figure 1 summarizes the command prior, residual policy, hazard critic, and deployable progression module.

### 3.1. System-Level Role and Workflow

ResCarry is an upper-level learning controller within a two-level humanoid control stack. The lower level is assumed to provide robot-state feedback, contact sensing, joint servoing, and scene/object-state estimates; in this simulation study, those quantities come from Isaac Lab or Unitree MuJoCo rather than from cameras or a hardware perception pipeline. The upper level receives proprioception, contact flags, a pose/identifier task tuple, and structured object pose/twist. It does not solve simultaneous localization and mapping, visual object recognition, or low-level torque stabilization. Its output is a sequence of whole-body joint-reference corrections around a nominal reference, which is then tracked by proportional–derivative (PD) joint control. This separation is consistent with common humanoid software stacks in which navigation, state estimation, scene perception, and joint-level feedback remain independent subsystems.

**Figure 2:**
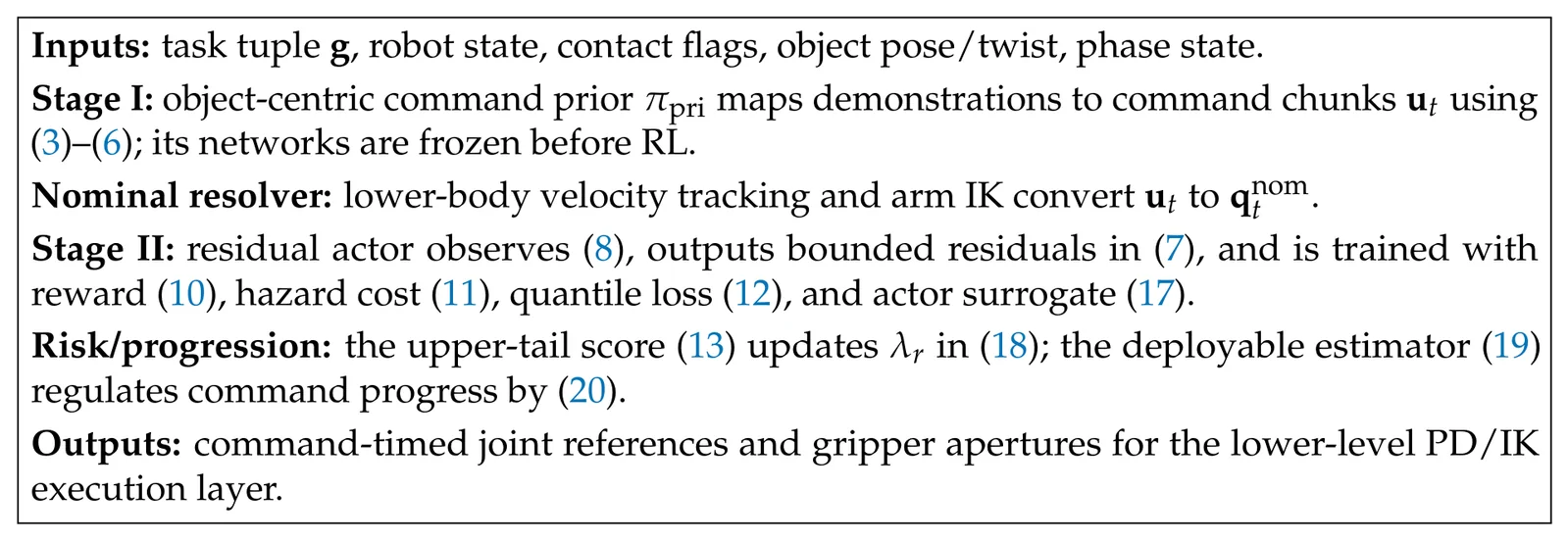
Workflow connecting the main equations to the two-stage training and deployment sequence. The diagram separates simulator/perception-side state provision, fixed command-prior generation, residual whole-body execution, and deployable hazard-based command progression.

The formal problem considered here is therefore not general autonomous scene understanding. Given a specified object, target region, contact mode, target yaw, and simulator-provided object-state, the problem is to generate and execute a dynamically feasible whole-body carrying behavior while reducing fall, drop, grasp-loss, collision, slip, and joint-limit hazards. Stage I learns the reference generator from retargeted demonstrations. Stage II keeps that reference generator fixed and learns the contact-conditioned residual execution policy. The relationship among the main variables, equations, and deployment roles is summarized in Figure 2.

### 3.2. Problem Setting and Hazard-Cost Formulation

We consider a single Unitree G1 humanoid equipped with two simple parallel grippers performing pick–carry–place tasks. Every episode is pose/identifier-specified: the manipulated object identifier $j^{★}$, the target-region identifier $k^{★}$, the contact mode $m^{★}\in \left\{\mathrm{btp},\mathrm{sh},\mathrm{sp},\mathrm{uep},\mathrm{bh}\right\}$ (back-top press, seat hold, side push, under-edge pull, bilateral hug), and the desired terminal object yaw $\psi^{★}$ are given directly as part of the task configuration. This specification,

(1) $$g=\left(j^{★},k^{★},m^{★},\psi^{★}\right),$$

is the same structured tuple used in prior grounded loco-manipulation work, together with the object pose $T_{O,t}$ and target pose $T_{G}$. Throughout, transformations written without a left superscript are expressed in the world frame *W*; a left superscript (e.g., ^*O*^(·) or ^*B*^(·)) denotes the object- or base-fixed frame.

The actor does not receive camera images. Object pose, twist, and task identifiers come from the simulator task configuration, with bounded latency and Gaussian position noise as part of the domain randomization (DR) described in Section 4; mass, friction, and contacts are randomized as well. This is a simulator-provided structured measurement interface, not a camera-based or learned object-state estimator.

We use a discounted partially observed control formulation with an auxiliary hazard cost,

(2) $$M=\langle S,O,A,P,r,c,\gamma ,\gamma_{c}\rangle ,$$

where S is the privileged simulator state, O is the deployable observation space, $A\subset R^{29}$ is the normalized residual action, *P* is the transition kernel, *r* is the task reward, c∈[0,1] is the hazard cost, and γ and $\gamma_{c}$ are reward and cost discount factors. Stage II uses an *approximate* Lagrangian relaxation of a tail-risk constraint. The actor objective combines reward and expected-cost advantages; the latter coefficient follows a projected dual-ascent step driven by an upper-tail (empirical CVaR) statistic of the hazard critic (Section 3.4).

The tracker requires a reference trajectory. A small secondary command prior (Section 3.3) provides a realistic, temporally structured, multi-phase reference. The reference is a 19-dimensional object-centric command,

(3) ut=vB,t⊤O,ψ˙B,tO,hcom,t,θtor,t,ΔξL,tO⊤,ΔξR,tO⊤,gL,t,gR,t⊤∈R19,

where ^*O*^$v_{B,t}\in R^{2}$ is planar base velocity expressed in the object yaw frame, ${\dot{\psi }}_{B,t}^{O}$ is relative base yaw rate, $h_{\mathrm{com},t}$ and $\theta_{\mathrm{tor},t}$ are center-of-mass height and torso pitch commands, $\Delta \xi_{h,t}^{O}\in R^{6}$ is the translational/rotational wrist pose increment for hand h∈{L,R} relative to an object-fixed grasp anchor determined by $m^{★}$, and $g_{L,t},g_{R,t}\in \left[0,1\right]$ are normalized gripper apertures. The corresponding desired wrist pose is

(4) TEh,t★=TO,tTAhOexpΔξh,tO^,

where $T_{O,t}\in \mathrm{SE}\left(3\right)$ is the world-frame object pose, TAhO is the fixed grasp anchor of hand *h* in the object frame, exp:se(3)→SE(3) is the matrix exponential, and $\hat{\left(\cdot \right)}:R^{6}\to \mathrm{se}\left(3\right)$ maps a twist to its matrix representation. Object-frame commands make the reference invariant to global scene translation and yaw, independent of whether the reference is produced by a learned prior or a hand-written trajectory. A fixed, separately trained lower-body velocity tracker and a damped-least-squares arm inverse kinematics (IK) solver then convert $u_{t}$ into a nominal whole-body reference $q_{t}^{\mathrm{nom}}=G\left(u_{t},q_{t}\right)\in R^{29}$ (Appendix C), around which Stage II learns bounded joint-reference corrections.

### 3.3. Stage I: Contact-Phase Command Prior

The command prior supplies $u_{t}$ from OMOMO-retargeted G1 demonstrations, giving the residual tracker a realistic multi-phase reference. In this paper, object-centric command prior has a narrow control meaning rather than the visual-recognition meaning of object-centric representation learning: the prior is a learned short-horizon command generator whose inputs and outputs are expressed relative to the manipulated object and its contact anchors. For example, wrist increments are represented in the object frame, base velocity is expressed in the object yaw frame, and phase transitions depend on measured contact and object progress. This representation lets the same learned command pattern translate with the object and target instead of being tied to a fixed world-frame trajectory.

Starting from OMOMO human–object interaction (HOI) sequences [8] retargeted to G1 [9], we form a pose/identifier-specified demonstration set $D={\left\{(g^{\left(n\right)},x_{1:T_{n}}^{\left(n\right)},u_{1:T_{n}}^{\left(n\right)},p_{1:T_{n}}^{\left(n\right)})\right\}}_{n=1}^{N_{D}}$, augmented by a privileged teacher for coverage as described in Appendix B. Here, $x_{t}$ contains object-relative robot and target geometry without image features, $u_{t}$ is the command in (3), and $p_{t}$ is the contact phase in P={app,sec,lift,trn,plc} (approach, secure, lift, transport, place). Commands are sampled at a fixed control rate and divided into non-overlapping *H*-step chunks $U^{p}\in R^{H\times 19}$ that never cross a phase boundary (values in Section 4).

Each phase *p* is assigned an encoder $E_{\phi }^{p}$, decoder $D_{\psi }^{p}$, and codebook $E^{p}={\left\{e_{k}^{p}\right\}}_{k=1}^{K_{\mathrm{cb}}}$ of latent dimension $d_{z}$. A chunk is encoded to $z=E_{\phi }^{p}\left(U^{p}\right)$ and quantized to $k^{★}=\arg\min_{k}{∥z-e_{k}^{p}∥}_{2}^{2}$, $z_{q}=e_{k^{★}}^{p}$. Following vector quantization (VQ) representation learning [34], the tokenizer loss is

(5) $$L_{\mathrm{VQ}}={∥M⊙W_{p}\left(U^{p}-D_{\psi }^{p}\left(z_{q}\right)\right)∥}_{1}+{∥\mathrm{sg}\left[z\right]-e_{k^{★}}^{p}∥}_{2}^{2}+\beta_{\mathrm{cmt}}{∥z-\mathrm{sg}[e_{k^{★}}^{p}]∥}_{2}^{2}-\beta_{\mathrm{ent}}\;H\left({\bar{p}}^{p}\right),$$

where M is a padding mask, $W_{p}$ a diagonal phase-specific command weight, sg the stop-gradient operator, $\beta_{\mathrm{cmt}}$ and $\beta_{\mathrm{ent}}$ are scalar coefficients, and $H({\bar{p}}^{p})$ is an entropy bonus on empirical code usage that discourages codebook collapse. A discrete action token is the pair $z_{n}=\left(p_{n},k_{n}\right)$.

A causal transformer with self-attention [35] then predicts the next phase-compatible token from a task-conditioning token (object/target dimensions, contact mode, terminal yaw), a geometry token (object-relative robot and target state), and a finite action token history; no visual token is used. The generator is trained with

(6) $$L_{\mathrm{IL}}=-\sum_{n}\log P_{\omega }\;\left(k_{n}|k_{<n},p_{n},g,x_{n}\right)+\beta_{\mathrm{ep}}{∥{\hat{y}}_{n+1}-y_{n+1}∥}_{2}^{2},$$

where the second term is an auxiliary object-relative endpoint prediction that discourages token sequences whose decoded effect drifts over a chunk. At inference, greedy decoding selects one token per exhausted chunk from the currently active phase codebook; consecutive chunks are blended with a short half-cosine window, and the phase automaton advances only when its measured transition condition is met (Appendix B). The tokenizer, transformer, and decoders are trained once and then frozen before residual tracker training; they are never updated by the reinforcement learning objective, so the reference $u_{t}$ seen by the tracker is fixed and reproducible across ablations.

**Figure 3:**
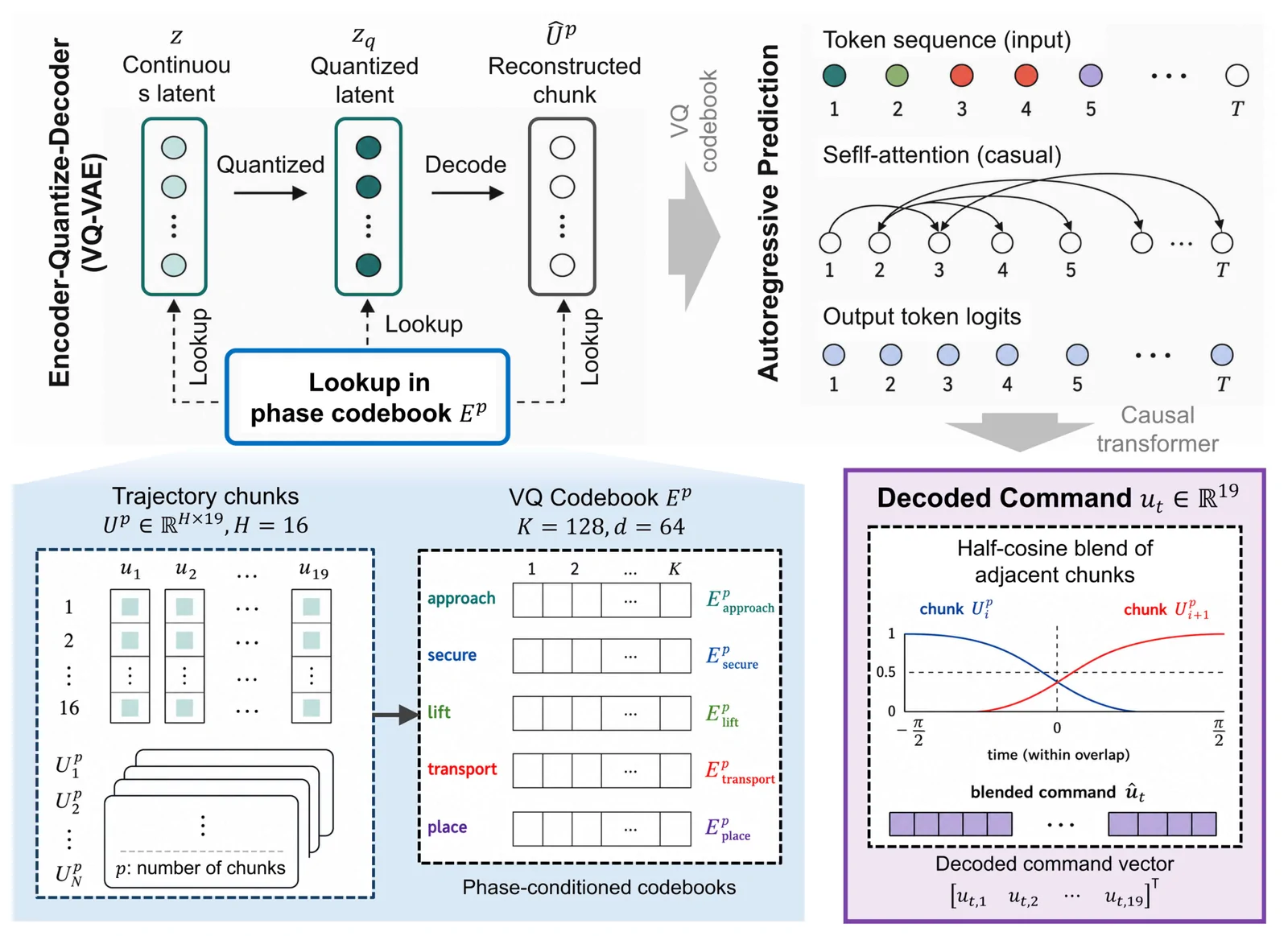
Contact-phase command prior (secondary component). Demonstrations are transformed to the object frame and segmented into approach, secure, lift, transport, and place phases, each with its own vector-quantized codebook. A causal transformer predicts phase-compatible tokens from the pose/identifier task tuple, object-relative geometry, and token history; the decoder reconstructs a smooth command chunk $u_{t}$ that is handed to the hazard-weighted residual tracker of Section 3.4 and Section 3.5.

Figure 3 summarizes the phase-conditioned tokenization and command-generation process.

### 3.4. Stage II: Residual Tracking with Quantile-Based Hazard Weighting

Stage II trains a residual whole-body policy that tracks $u_{t}$ and reduces the hazard indicators defined below. Residual limits, reward/hazard weights, and learning rates appear in Section 4 and Appendix C. The tracker outputs a normalized residual $a_{t}\in {\left[-1,1\right]}^{29}$ around the nominal reference:   

(7) $$q_{t}^{\mathrm{cmd}}=q_{t}^{\mathrm{nom}}+s_{a}⊙\tanh\left(a_{t}\right),\;\tau_{t}=K_{p}\left(q_{t}^{\mathrm{cmd}}-q_{t}\right)-K_{d}{\dot{q}}_{t},$$

where $s_{a}\in R_{>0}^{29}$ is a vector of per-joint residual amplitude limits, with entries grouped by legs, waist, and arms. Gripper apertures are taken directly from $u_{t}$ and are not part of the 29-dimensional residual. The deployable observation is

(8) otπ=qt−qtnom,q˙t,ωB,t,gB,t,vB,t,ut,ξO,tB,vO,tB,bcnt,t,at−1,oh(pt),st∈Rdπ,

where $g_{B,t}$ is projected gravity, ξO,tB∈R6 is object pose relative to the base (3-D position + axis-angle), vO,tB∈R6 is the corresponding twist, $b_{\mathrm{cnt},t}\in {\left\{0,1\right\}}^{4}$ are foot/gripper contact flags (simulator-idealized binary switches), and $s_{t}\in \left[0,1\right]$ is progress through the active chunk. Object pose and twist enter only through this structured interface (Section 3.2). Training uses a separate critic input

(9) $$o_{t}^{c}=\left[o_{t}^{\pi },\xi_{t}^{\mathrm{priv}}\right],$$

where $\xi_{t}^{\mathrm{priv}}$ stacks privileged simulator fields (true mass, friction, inertia and CoM offsets, DR latency parameters, external wrench, and contact forces) that are unavailable on hardware and are not used by the actor or deployable hazard estimator.

The task reward combines reference tracking, phase-specific object progress, grasp maintenance, posture, and control regularity:

(10) $$\begin{array}{cc} r_{t} & =w_{\mathrm{bas}}\;r_{\mathrm{bas}}+w_{\mathrm{wri}}\;r_{\mathrm{wri}}+w_{\mathrm{obj}}\;r_{\mathrm{obj}}+w_{\mathrm{grp}}\;r_{\mathrm{grp}}+w_{\mathrm{up}}\;r_{\mathrm{up}}\\ \; & \;-w_{\tau }∥\tau_{t}⊙{\dot{q}}_{t}{∥}_{1}-w_{\Delta a}∥a_{t}-a_{t-1}{∥}_{2}^{2}-w_{\ddot{q}}{∥{\ddot{q}}_{t}∥}_{2}^{2}, \end{array}$$

where each positive term lies in [0,1] (an exponential of a nonnegative quadratic error) and the nonnegative weights $w_{\left(\cdot \right)}$ are fixed during Stage II. Boundedness of $r_{t}$ is all that the subsequent analysis requires.

The instantaneous hazard cost is defined as the maximum of the weighted failure indicators,

(11) $$c_{t}=\max\left\{w_{\mathrm{fal}}1_{\mathrm{fal}},\;w_{\mathrm{drp}}1_{\mathrm{drp}},\;w_{\mathrm{gl}}1_{\mathrm{gl}},\;w_{\mathrm{col}}1_{\mathrm{col}},\;w_{\mathrm{slp}}1_{\mathrm{slp}},\;w_{\lim}1_{\lim}\right\}\in \left[0,1\right],$$

with binary detectors for fall (fal), drop (drp), grasp loss (gl), collision (col), stance slip (slp), and joint-limit proximity (lim; thresholds in Appendix C). The maximum defines a worst-case hazard model: one severe failure at time *t* dominates $c_{t}$. A weighted sum would let minor events accumulate to the magnitude of a catastrophic event.

To represent the distribution of discounted hazard, a quantile critic estimates $Z_{t}^{c}=\sum_{\ell \geq 0}\gamma_{c}^{\ell }c_{t+\ell }$ through *M* outputs ${\left\{z_{m}\left(o_{t}^{c}\right)\right\}}_{m=1}^{M}$ at fixed levels $\tau_{m}=\left(m-0.5\right)/M$. Episode termination and rollout truncation are handled by the bootstrap mask $d_{t}$. With target $y_{j}=c_{t}+\gamma_{c}\left(1-d_{t}\right)z_{j}^{-}\left(o_{t+1}^{c}\right)$, the critic is trained by quantile Huber regression [33],

(12) $$L_{\mathrm{QR}}=\frac{1}{M^{2}}\sum_{i=1}^{M}\sum_{j=1}^{M}\rho_{\tau_{i}}^{\kappa }\;\left(y_{j}-z_{i}\left(o_{t}^{c}\right)\right),\;\rho_{\tau }^{\kappa }\left(u\right)=\left|\tau -1_{u<0}\right|\;H_{\kappa }\left(u\right),$$

where $H_{\kappa }$ is the Huber loss with threshold κ.

For a tail level α∈(0,1), let $K_{\alpha }=\max\left(1,\left\lceil \left(1-\alpha \right)M\right\rceil \right)$ and $M_{\alpha }=\left\{M-K_{\alpha }+1,\ldots ,M\right\}$ index the atoms at the $K_{\alpha }$ highest quantile levels. Because $\left\{z_{m}\left(o_{t}^{c}\right)\right\}$ are the quantile estimates of $Z_{t}^{c}$ at levels $\tau_{m}$, their tail average is the standard finite-atom empirical estimator of the conditional value-at-risk of the discounted hazard return:   

(13) $${\hat{\mathrm{CVaR}}}_{\alpha }\left(Z_{t}^{c}\right)\;\equiv \;{\hat{R}}_{t}^{\mathrm{tl}}\left(\alpha \right)=\frac{1}{|M_{\alpha }|}\sum_{m\in M_{\alpha }}z_{m}\left(o_{t}^{c}\right).$$

This upper-tail estimator becomes exact for the population ${\mathrm{CVaR}}_{\alpha }$ as M→∞ with $\alpha =1-K_{\alpha }/M$; at finite *M*, it is a quantile-discretized approximation [33]. We use it as the upper-tail hazard score. The deployed value of α is specified in Section 4; the joint risk/progression configuration comparison is reported in Section 5.4.

We formulate the hazard coefficient as the dual variable of the tail-risk-constrained policy optimization problem

(14) $$\max_{\theta }\;E\;\left[\sum_{t}\gamma^{t}r_{t}\right]\;s.t.\;E\;\left[{\hat{\mathrm{CVaR}}}_{\alpha }\left(Z_{t}^{c}\right)\right]\leq d_{r},$$

whose Lagrangian relaxation, with multiplier $\lambda_{r}\geq 0$, is

(15) $$L\left(\theta ,\lambda_{r}\right)=E\;\left[\sum_{t}\gamma^{t}r_{t}\right]-\lambda_{r}\left(E\;\left[{\hat{\mathrm{CVaR}}}_{\alpha }\left(Z_{t}^{c}\right)\right]-d_{r}\right).$$

The constraint is monitored by the batch estimate

(16) $${\bar{R}}_{\mathrm{tl}}=\frac{1}{\left|B\right|}\sum_{t\in B}{\hat{\mathrm{CVaR}}}_{\alpha }\left(Z_{t}^{c}\right).$$

For the primal step, we replace the intractable direct CVaR gradient by a clipped PPO update on a reward generalized advantage estimation (GAE) ${\hat{A}}_{t}^{r}$ and an expected-cost GAE ${\hat{A}}_{t}^{c}$ bootstrapped from the mean quantile value. Let $r_{t}^{\mathrm{imp}}=\pi_{\theta }\left(a_{t}|o_{t}^{\pi }\right)/\pi_{\theta^{-}}\left(a_{t}|o_{t}^{\pi }\right)$. The actor maximizes

(17) $$\begin{array}{cc} L_{\mathrm{act}}\left(\theta \right)\; & =E\;\left[\min\;\left(r_{t}^{\mathrm{imp}}{\hat{A}}_{t}^{r},\mathrm{clip}\left(r_{t}^{\mathrm{imp}},1-\epsilon ,1+\epsilon \right){\hat{A}}_{t}^{r}\right)\right]\\ \; & \;-\lambda_{r}\;E\;\left[\max\;\left(r_{t}^{\mathrm{imp}}{\hat{A}}_{t}^{c},\mathrm{clip}\left(r_{t}^{\mathrm{imp}},1-\epsilon ,1+\epsilon \right){\hat{A}}_{t}^{c}\right)\right], \end{array}$$

with PPO clip ϵ>0. The reward term uses the usual lower clipped surrogate for maximization, whereas the cost term uses the upper clipped surrogate inside the penalty so that cost increases are not made artificially optimistic by the importance-ratio clip. The multiplier follows the corresponding projected dual-ascent step on (15),

(18) $$\lambda_{r}\leftarrow P_{\Lambda }\;\left(\lambda_{r}+\eta_{\lambda }\left({\bar{R}}_{\mathrm{tl}}-d_{r}\right)\right)={\left[\lambda_{r}+\eta_{\lambda }\left({\bar{R}}_{\mathrm{tl}}-d_{r}\right)\right]}_{+},$$

where ${\bar{R}}_{\mathrm{tl}}-d_{r}=-\nabla_{\lambda_{r}}L$ is the estimated constraint violation, $d_{r}$ is the reference tail level, $\eta_{\lambda }>0$ the dual step size, and the projection $P_{\Lambda }\left(x\right)=\max\left(0,x\right)$ onto $\Lambda =R_{\geq 0}$ keeps the multiplier nonnegative. This standard Lagrangian-PPO penalty schedule [30,31] raises $\lambda_{r}$ when the batch upper-tail hazard exceeds $d_{r}$ and relaxes it otherwise. The update remains approximate because the primal step is a clipped expected-cost PPO surrogate and $\pi_{\theta }$ is non-convex. We call the procedure quantile-based adaptive hazard weighting (hazard-weighted RL). Hyperparameters and the GAE definition appear in Appendix C.

### 3.5. Adaptive Command Progression

The privileged hazard critic is unavailable at deployment because it receives simulator-only quantities. We therefore train a deployable estimator $\rho_{\chi }\left(o_{t}^{\pi }\right)\in \left[0,1\right]$ using the actor observation,

(19) $$L_{\mathrm{stu}}={∥\rho_{\chi }\left(o_{t}^{\pi }\right)-\mathrm{clip}\;\left({\hat{R}}_{t}^{\mathrm{tl}}/Z_{\max}^{c},0,1\right)∥}_{2}^{2}+\beta_{\mathrm{bce}}\;\mathrm{BCE}\;\left(\rho_{\chi }\left(o_{t}^{\pi }\right),y_{t}^{H_{\mathrm{evt}}}\right),$$

where $y_{t}^{H_{\mathrm{evt}}}$ indicates whether a hazard event occurs within the next $H_{\mathrm{evt}}$ control steps, $Z_{\max}^{c}$ clips the upper-tail target, and $\beta_{\mathrm{bce}}\geq 0$ balances the two terms. $\rho_{\chi }$ combines the normalized upper-tail target with a near-term event label, so it is not a pure event probability. Gradients from $L_{\mathrm{stu}}$ do not flow into the actor. Appendix C details the reporting-only calibration diagnostics (temperature scaling on pre-sigmoid logits and expected calibration error (ECE)).

The estimated score $\rho_{\chi ,t}$ regulates progress $\zeta_{t}\in \left[0,H-1\right]$ through the currently active command chunk,

(20) $$\begin{array}{cc} \zeta_{t+1} & =\min\;\left[H-1,\;\zeta_{t}+\Delta \zeta \;\kappa \left(\rho_{\chi ,t}\right)\right],\\ \kappa (\rho ) & =\mathrm{clip}\;\left(\frac{\rho_{\mathrm{hld}}-\rho }{\rho_{\mathrm{hld}}-\rho_{\mathrm{slw}}},\;0,\;1\right), \end{array}$$

where Δζ converts the residual control rate to command-chunk progress, and $\rho_{\mathrm{slw}}<\rho_{\mathrm{hld}}$ define the slow-down and hold thresholds. The progression rate κ(ρ) equals one below $\rho_{\mathrm{slw}}$, decreases linearly between the thresholds, and reaches zero at $\rho_{\mathrm{hld}}$. Linear interpolation between neighboring command samples yields $u(\zeta_{t})$. When $\rho_{\chi ,t}\geq \rho_{\mathrm{hld}}$, commanded base velocity is set to zero and the last object-relative wrist targets are held as the residual policy recovers balance. Progression resumes once the score remains below $\rho_{\mathrm{res}}$ for $n_{\mathrm{res}}$ consecutive control steps. High estimated hazard therefore slows or stops reference advancement. The rule changes only command timing and is evaluated against open-loop progression.

Appendix A relates the finite-atom empirical estimator to population CVaR and summarizes the dual-ascent interpretation of (18).

Algorithm 1 is organized around the two-stage separation used throughout the paper. Lines 1–2 train the motion-reference generator only from demonstration commands and then freeze it. Lines 3–11 train the residual execution layer: the actor observes deployable state, the reward and hazard streams train separate critics, and the quantile critic supplies the upper-tail statistic used to adapt $\lambda_{r}$. Line 12 trains the deployable hazard estimator from actor-side observations only, so the privileged critic is not required after training. Lines 14–16 describe the exported deployment loop: the frozen prior produces the next object-centric command, the estimator decides whether command time should advance or pause, and the residual policy sends bounded joint-reference corrections to the lower-level controller.
**Algorithm 1** ResCarry Training and Deployment.**Require:** Demonstrations D; phase rules P; Isaac Lab environment; upper-tail level α, reference value $d_{r}$  1:Train phase-conditioned tokenizer codebooks $\left(E_{\phi }^{p},D_{\psi }^{p},E^{p}\right)$ using (5) for every p∈P  2:Train the causal token transformer using (6); freeze the tokenizer and transformer  3:Initialize residual policy $\pi_{\theta }$, value critic, quantile hazard critic, and deployable hazard estimator $\rho_{\chi }$  4:**for** each PPO iteration **do**  5:    Sample pose/identifier tasks, payload, contact, latency, and perturbation parameters  6:    Decode the active-phase command $u_{t}$ from the frozen command prior  7:    Execute the residual controller (7) under adaptive progression (20) driven by the online hazard score estimator  8:    Store rewards $r_{t}$, hazard costs $c_{t}$, privileged state, and deployable observations  9:    Update the value critic and the quantile hazard critic using (12) 10: Compute the upper-tail score via (13), form cost GAE ${\hat{A}}_{t}^{c}$, and update $\pi_{\theta }$ with the actor surrogate (17) 11:    Update the penalty coefficient $\lambda_{r}$ via (18) 12:    Train the deployable hazard estimator with (19) 13:**end for** 14:After PPO: fit temperature scaling on held-out event labels for the calibration diagnostic only 15:Export the residual actor, hazard estimator, command-prior networks, and observation normalizer to ONNX 16:Deploy: at each control step, decode $u(\zeta_{t})$, compute $\rho_{\chi ,t}$, update $\zeta_{t+1}$ via (20), and apply (7) 17:Evaluate the unchanged networks in Unitree MuJoCo without any parameter update

## 4. Experimental Evaluation

Policies are trained in Isaac Lab on the official Unitree G1 29-DoF assets [24,25] and transferred without parameter updates to Unitree MuJoCo [26,27]. The actor observation is identical across engines and consists of proprioception, contact flags, and structured object pose/twist (Section 3.2). Evaluation covers six everyday loco-manipulation skills with explicit hand–object contact geometry: (T1) Chair-drag—hands press the top of the chair back and drag; (T2) Chair-carry—hands hold the seat and lift-carry; (T3) Suitcase-push—bent torso, push the suitcase on a vertical side face; (T4) Storage-push—bent torso, push a storage box on a side face; (T5) Table-pull—bent torso, under-edge fingertip grip, pull the table; (T6) Bin-carry—bilateral hug on a trash bin and carry. Each episode requires locomotion under sustained contact and at least one heading change.

Evaluation uses the following metrics and protocol. Success (SR): An episode is successful if the object reaches the goal region with terminal yaw error below the task tolerance, the base remains upright without a fall or drop event, and the episode ends within the phase timeout (Appendix B); otherwise it counts as failure. SR is the fraction of successful episodes, computed per seed and task then averaged across seeds. Hazard rate: Fraction of evaluation episodes in which at least one instantaneous hazard in (11) is active (episode-level indicator; not a time-step average of $c_{t}$). Tracking errors $E_{o}$, $E_{m}$, $E_{j}$: Time-weighted root-mean-square (RMS) errors between commanded and realized object pose, end-effector motion, and joint references, averaged over the full episode (including failures unless noted). Checkpoints: Each seed and method exports the final weights after the fixed Stage II budget ($2.4\times 10^{8}$ environment steps, PPO cap $6\;\times \;10^{3}$); no test-episode checkpoint selection. Evaluation rollouts: The primary Isaac and MuJoCo success tables use 40 evaluation episodes per task, seed, method, and simulator, i.e., 200 binary episodes per task–method cell and 1200 episodes per method for a six-task mean. Ablation and risk-configuration rows use the same 40 episodes per task and seed. Added-payload stress rows use 30 episodes per mass, seed, method, and task. Calibration diagnostics use 12,800 held-out transition pairs. Random seeds, task tuples, initial robot/object poses, randomized masses/frictions/latencies, and perturbation schedules are matched across methods within each simulator before applying method-specific policy actions. Per-task table entries are seed-averaged percentages reported to 0.5 pp resolution where applicable. Mass-stress Fail: At that total mass, the success criterion is not met for any evaluation episode in the batch (tracking errors are then omitted).

Simulation is configured as follows. Isaac Lab uses the official Unitree G1 articulation with 29 actuated joints, GPU PhysX, a 0.005 s physics step, and policy decimation to 50 Hz. The lower-level PD loop receives joint targets from (7); self-collisions and rigid-body contacts use the simulator defaults for the official asset except for the task-specific object mass/friction randomization specified below. Unitree MuJoCo uses the official G1 model with the same actor observation layout, PD gains, command interface, task initializers, and success definitions. The transfer experiments do not retune gains, randomization ranges, normalizers, or neural-network weights in MuJoCo.

Unless noted, all residual comparisons share the reward terms, observation $o_{t}^{\pi }$, nominal resolver G, and domain randomization specified below. Base prior uses fixed Stage I commands with IK/proportional–derivative (PD) only. Coarse-reference residual uses residual PPO with the same observation and G, but a hand-written phase schedule in place of the learned prior. Continued supervised prior training updates the command prior only with the Stage I supervised loss on replayed command data; PPO gradients do not pass through greedy token selection. PhaseIL–PPO uses the fixed prior with a fixed expected-cost coefficient $\lambda_{r}$ and open-loop progression. ResCarryadds quantile-based adaptive $\lambda_{r}$ and the deployable progression module under the shared curriculum.

To avoid ambiguous interpretation of the comparison, all reported methods are evaluated with the same success definitions, timeout limits, randomized task tuples, object meshes, and perturbation schedules. We do not select the best checkpoint using test episodes, and we do not tune a method separately for MuJoCo after Isaac training. The statistical table therefore treats each method as a fixed exported policy under the declared evaluation protocol. Percentages in the main tables should be read as finite-sample simulation estimates, not as claims of hardware reliability or unrestricted generalization outside the structured object-state setting.

Stage I uses N=2400 trajectories (400 per task): 300 OMOMO-retargeted clips and 100 teacher-generated clips per task. Commands are sampled at 20 Hz with chunk length H=16; each phase codebook has $K_{\mathrm{cb}}\;=\;128$ entries of dimension $d_{z}\;=\;64$; the causal transformer has eight layers, width 256, and context length 32; VQ/IL coefficients are $\left(\beta_{\mathrm{cmt}},\beta_{\mathrm{ent}},\beta_{\mathrm{ep}}\right)=\left(0.25,0.01,0.2\right)$ (Appendix B). Stage II uses 4096 parallel Isaac Lab environments, a 50 Hz residual policy, and $2.4\times 10^{8}$ environment steps × five seeds. Deployable observation dimension $d_{\pi }\;=\;137$; residual limits $s_{a}$ are 0.20/0.25/0.30 rad (legs/waist/arms). Reward weights are $\left(w_{\mathrm{bas}},w_{\mathrm{wri}},w_{\mathrm{obj}},w_{\mathrm{grp}},w_{\mathrm{up}}\right)=\left(1.2,1.5,1.0,0.8,0.5\right)$ with penalties $\left(w_{\tau },w_{\Delta a},w_{\ddot{q}}\right)=\left(2\times 10^{-5},0.01,0.02\right)$. Hazard indicator weights are $\left(w_{\mathrm{fal}},w_{\mathrm{drp}},w_{\mathrm{gl}},w_{\mathrm{col}},w_{\mathrm{slp}},w_{\lim}\right)=\left(1,1,0.8,0.6,0.4,0.3\right)$. Quantile critic: M=32, $\gamma_{c}\;=\;0.97$, deployed α=0.9 ($K_{\alpha }\;=\;4$), PPO clip ϵ=0.2, reference $d_{r}\;=\;0.25$, dual step $\eta_{\lambda }\;=\;5\;\times \;10^{-3}$; PhaseIL–PPO uses fixed $\lambda_{r}\;=\;0.12$. Progression: Δζ=20/50, $\left(\rho_{\mathrm{slw}},\rho_{\mathrm{hld}}\right)=\left(0.15,0.45\right)$, resume $\left(\rho_{\mathrm{res}},n_{\mathrm{res}}\right)=\left(0.25,10\right)$, student $\left(Z_{\max}^{c},\beta_{\mathrm{bce}},H_{\mathrm{evt}}\right)=\left(8,0.2,50\right)$. Detector thresholds and PD gains are in Appendix C; network architectures are summarized in Table 1.

Table 2 summarizes the six contact-specified tasks and their randomized mass and travel ranges.

Table 3 lists the domain-randomization ranges used during residual training.

## 5. Results

### 5.1. Comparison with Reference and Training Variants

**Figure 4:**
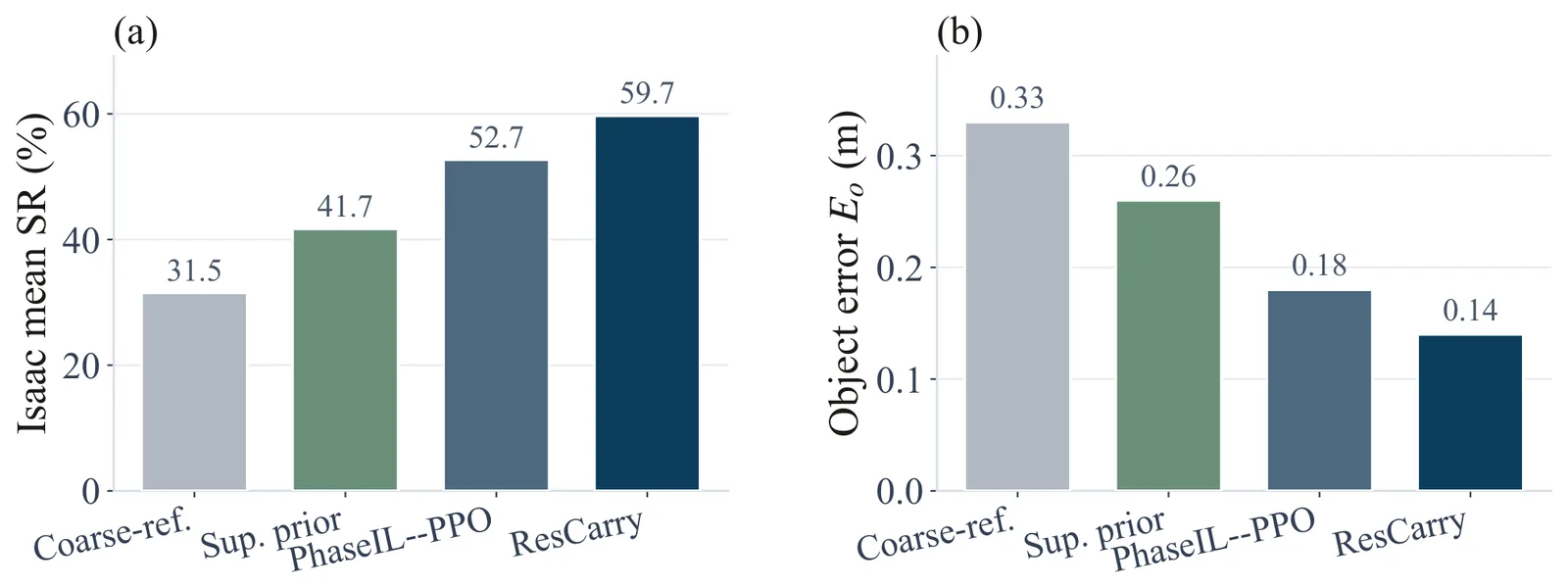
Visual summary of the primary Isaac Lab comparison. (**a**) Six-task mean success. (**b**) Mean object tracking error $E_{o}$. Axis labels abbreviate the baselines of Table 4: Coarse-ref. = coarse-reference residual; Sup. prior = continued supervised prior training.

The primary Isaac Lab comparison in Table 4 rises from 16.5% mean success for the fixed prior to 31.5% for the coarse-reference residual and 41.7% for continued supervised prior training. PhaseIL–PPO reaches 52.7%, with mean object error falling to 0.18 m. ResCarry improves further to 59.7% mean success with $E_{o}=0.14$ m, $E_{m}=0.50$ m, and $E_{j}=1.35$ rad. Table-pull has the lowest ResCarry success at 53%; Chair-carry and Bin-carry reach 66% and 65%. These are five-seed means. Figure 4 provides a visual summary of the primary Isaac Lab comparison. Table 5 adds pooled Wilson confidence intervals for the six-task success rates and two-proportion tests against ResCarry using the matched evaluation protocol described in Section 4.

**Figure 5:**
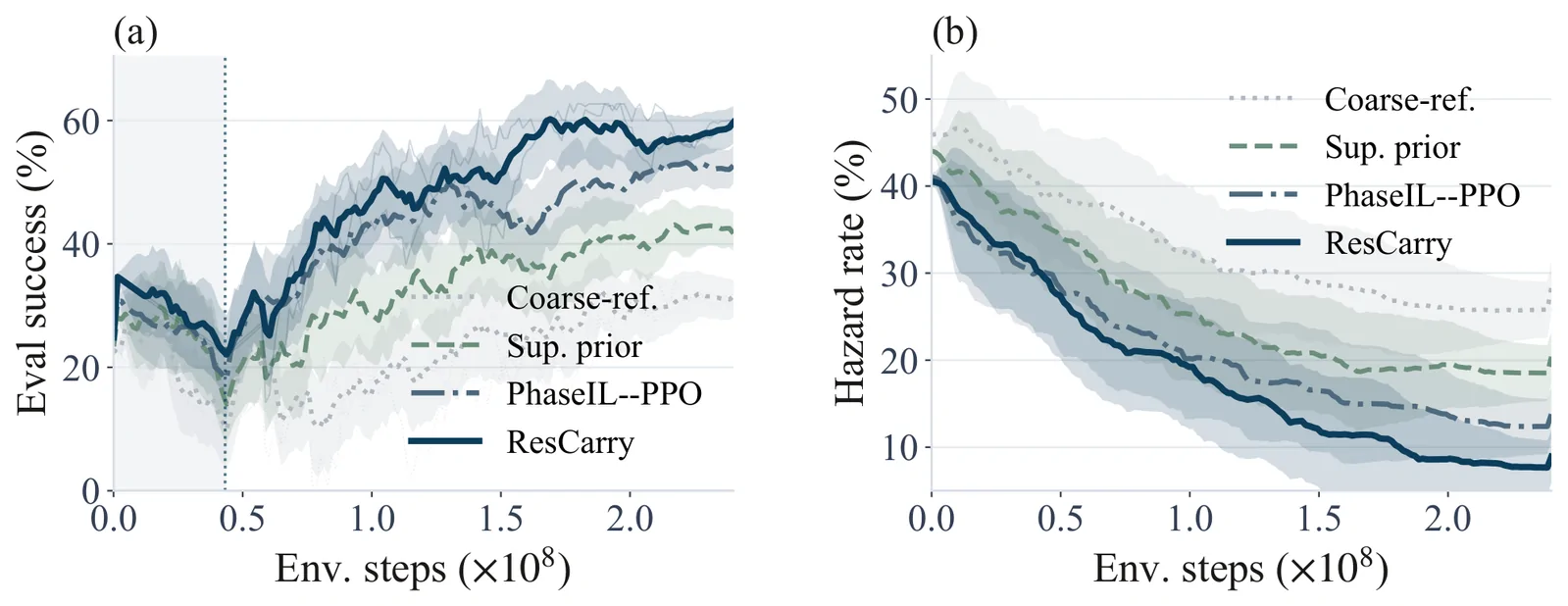
Isaac Lab Stage II training under a shared curriculum. Curves are checkpoint evaluations on the six-task suite (logged during training; five-seed mean with shaded mean ± one standard deviation). (**a**) Eval success (plateaus match Table 4). (**b**) Episode-level hazard rate. Legend abbreviations as in Figure 4.

**Figure 6:**
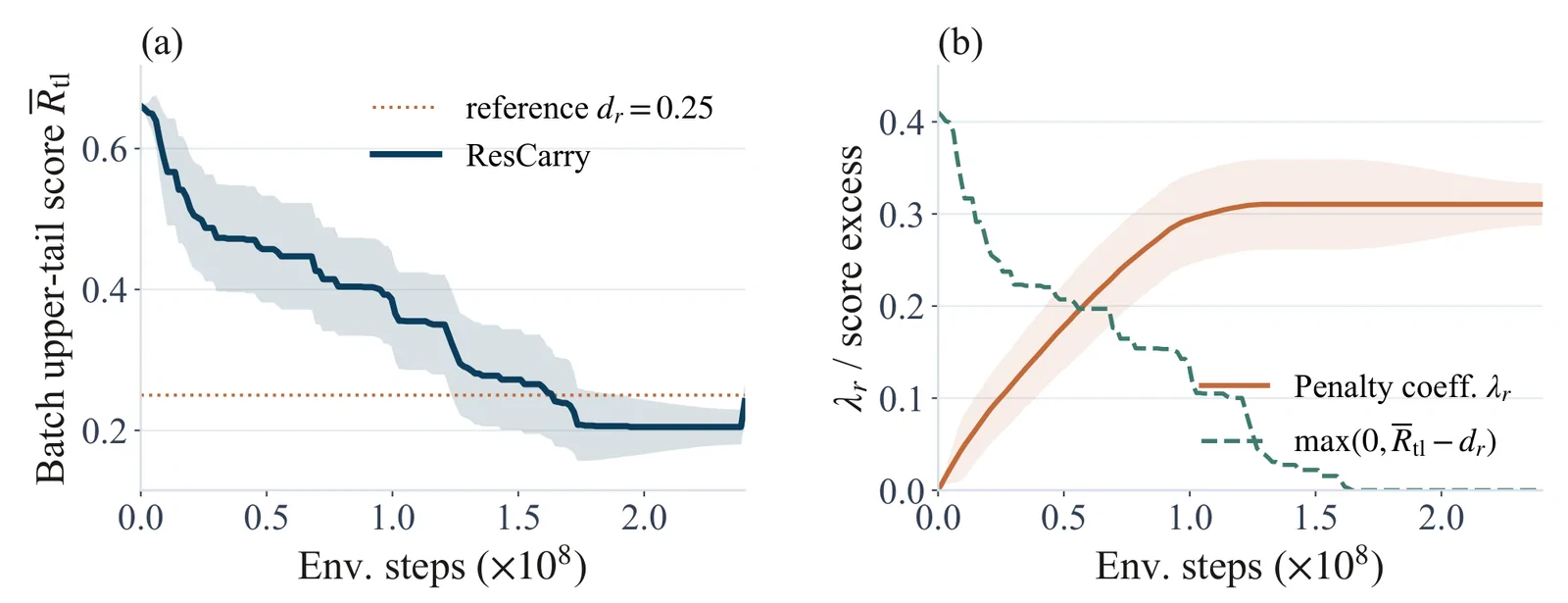
Upper-tail hazard weighting during Stage II training (ResCarry). Checkpoint evaluations on the six-task suite; five-seed mean ± std. (**a**) Batch upper-tail score ${\bar{R}}_{\mathrm{tl}}$ (dotted: reference $d_{r}=0.25$). (**b**) Penalty coefficient $\lambda_{r}$ and $\max(0,{\bar{R}}_{\mathrm{tl}}-d_{r})$.

Stage II learning under the shared standing → task curriculum is summarized in Figure 5, where the vertical marker denotes the end of the curriculum. Success and hazard are evaluated on the six-task suite throughout training, and the late plateaus align with the mean SR and hazard in Table 4 for each residual variant. For ResCarry, the evolution of the batch upper-tail score relative to $d_{r}$ and the associated adaptive coefficient $\lambda_{r}$ is reported in Figure 6.

**Figure 7:**
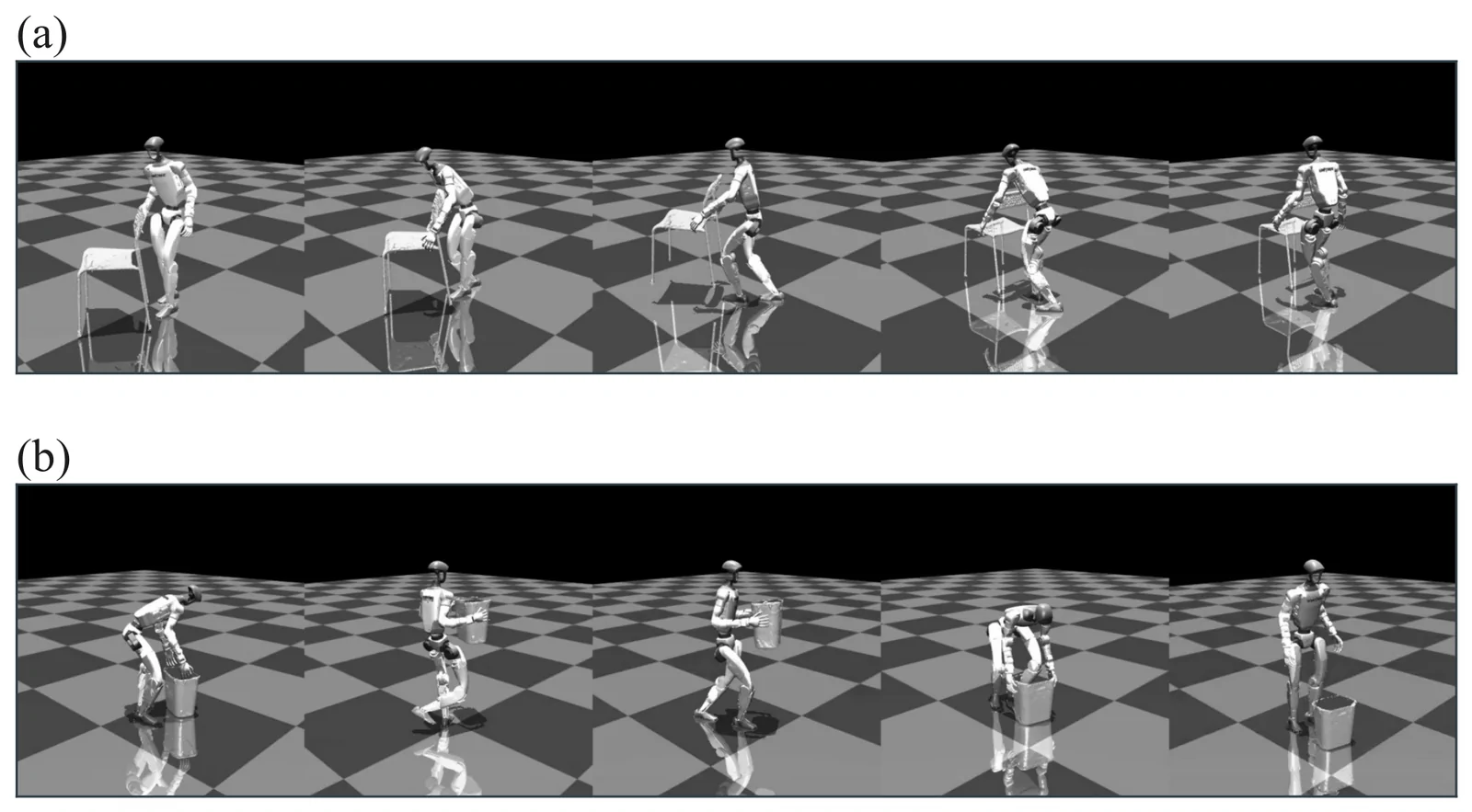
MuJoCo zero-update qualitative evidence for lift/carry contacts. (**a**) T2 Chair-carry (seat hold/lift-carry). (**b**) T6 Bin-carry (bilateral hug).

Fixing Stage I and training only the residual policy raises mean success by 18.0 points over continued supervised prior training (Table 4). The same controller maintains sustained seat hold and bilateral hug during the zero-update MuJoCo Chair-carry and Bin-carry rollouts in Figure 7.

### 5.2. Sim-to-Sim Object Fidelity

**Figure 8:**
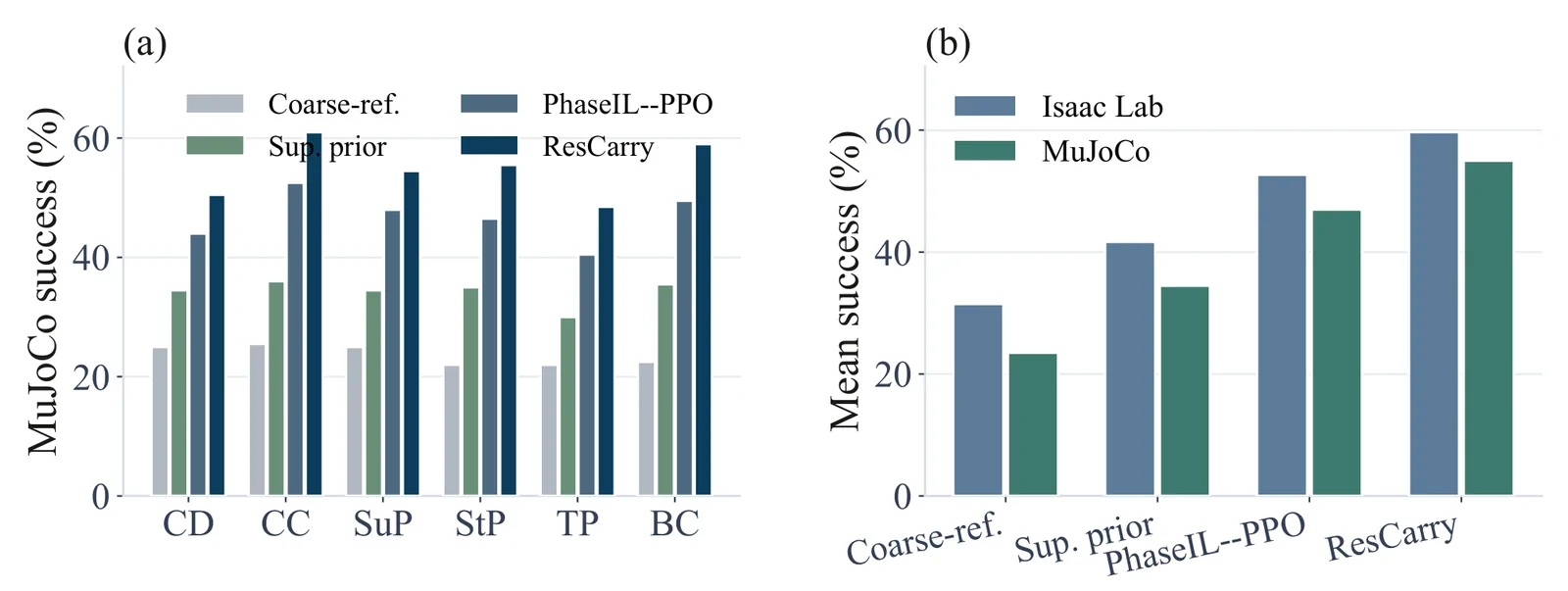
Visual summary of the zero-update MuJoCo evaluation. (**a**) Per-task success on the six furniture tasks. (**b**) Isaac vs. MuJoCo six-task means. Legend abbreviations as in Figure 4.

Object tracking error $E_{o}$ in Table 4 measures episode-level object-following performance in Isaac Lab. The zero-update MuJoCo success values in Table 6 and Figure 8 provide the corresponding cross-engine evaluation. ResCarry reaches mean $E_{o}=0.14$ m on Isaac and 54.8% overall MuJoCo SR versus 46.9% for PhaseIL–PPO, 34.3% for continued supervised prior training, and 23.7% for the coarse-reference residual; the Isaac → MuJoCo gap for ResCarry is 4.9 points. Table-pull remains hardest in MuJoCo, while Chair-carry/Bin-carry remain easiest—the same difficulty order as Isaac.

**Figure 9:**
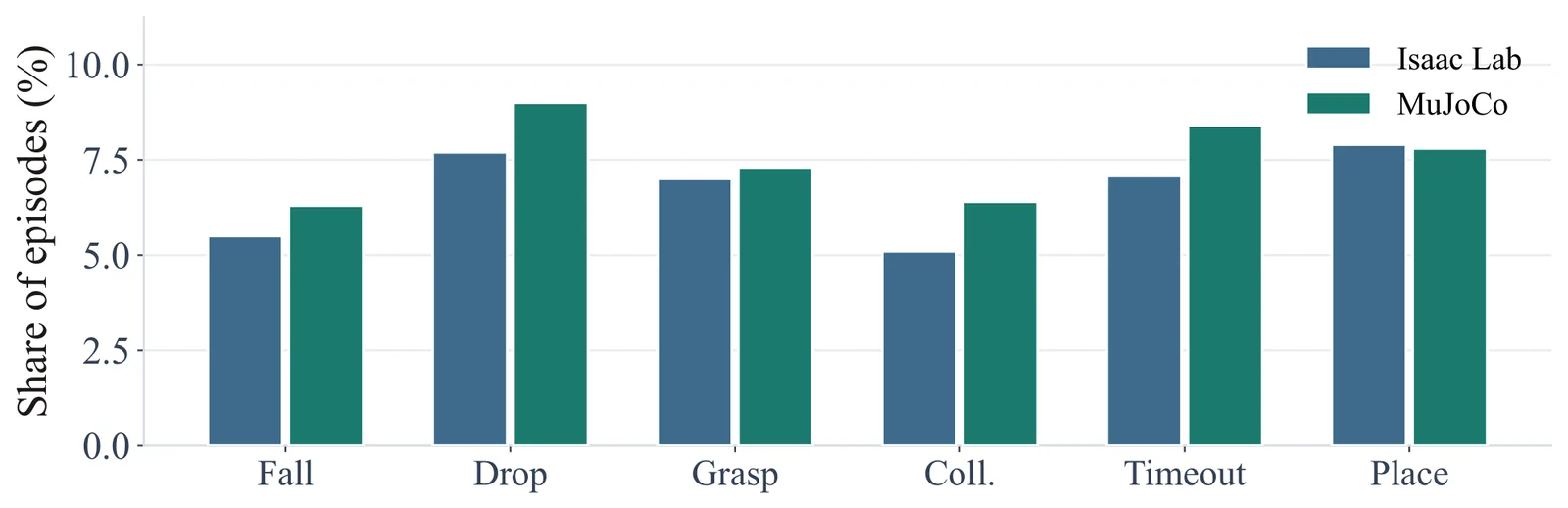
Failure taxonomy for the full method. Per-class episode shares in Isaac Lab and zero-update MuJoCo.

**Figure 10:**
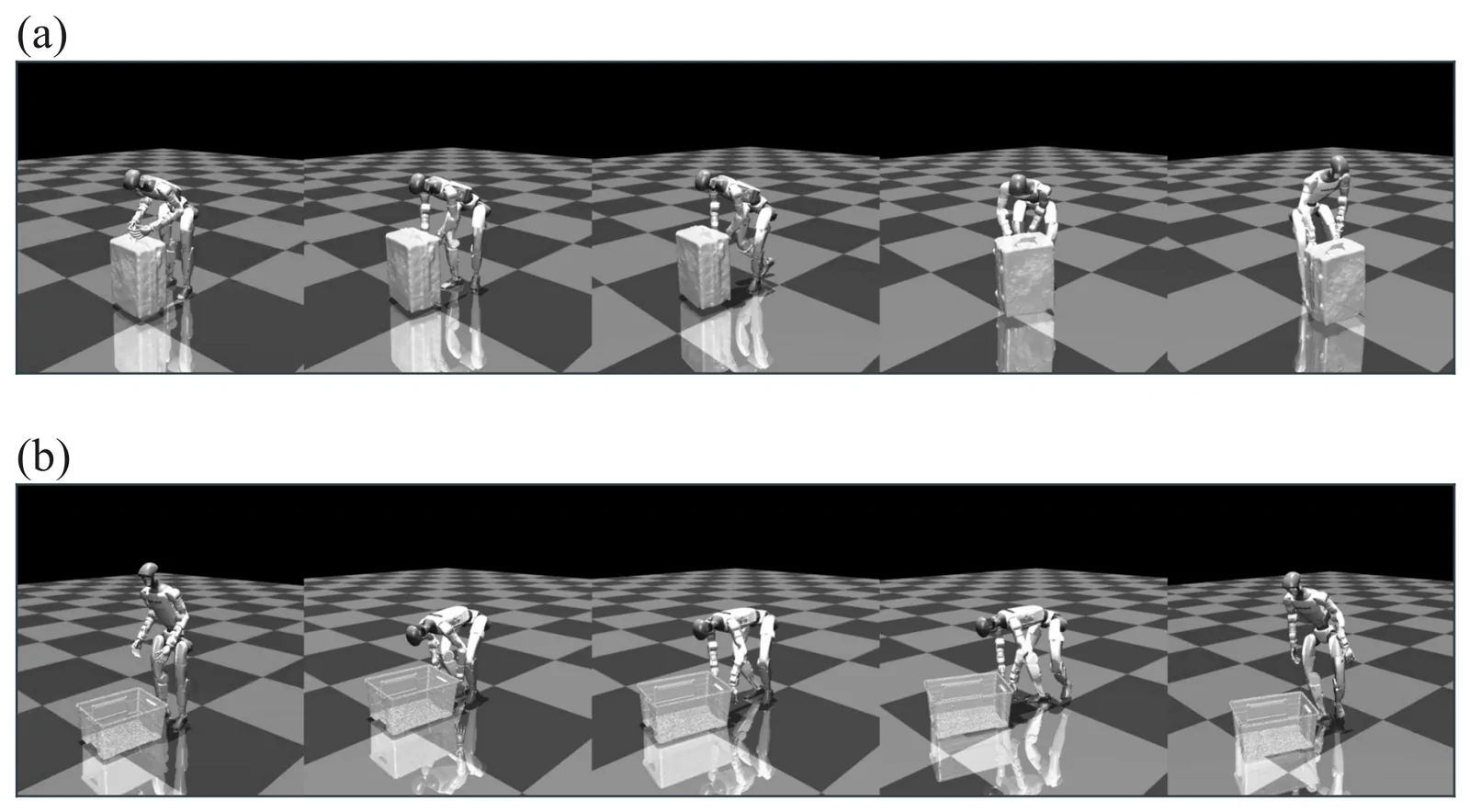
MuJoCo zero-update qualitative evidence for bent side-push contacts. (**a**) T3 Suitcase-push. (**b**) T4 Storage-push.

Table 7 and Figure 9 break down unsuccessful ResCarry episodes. MuJoCo increases grasp-loss and phase-timeout shares relative to Isaac; falls and collisions remain secondary. There is no perception/grounding failure class because tasks are pose/identifier-specified. In 72% of cases, high-risk student triggers precede torso tilt $>12^{\circ }$, one-sided contact loss, stance slip, and large object angular velocity within 0.6 s. On held-out, size- and grasp-class-matched meshes, success drops to 55.6%/52.2% (Isaac/MuJoCo). The drop is reported without attributing it to a particular module. Under the same zero-update MuJoCo policy, the Suitcase and Storage tasks retain the bent side-push contact pattern shown in Figure 10.

### 5.3. Component Ablations

**Figure 11:**
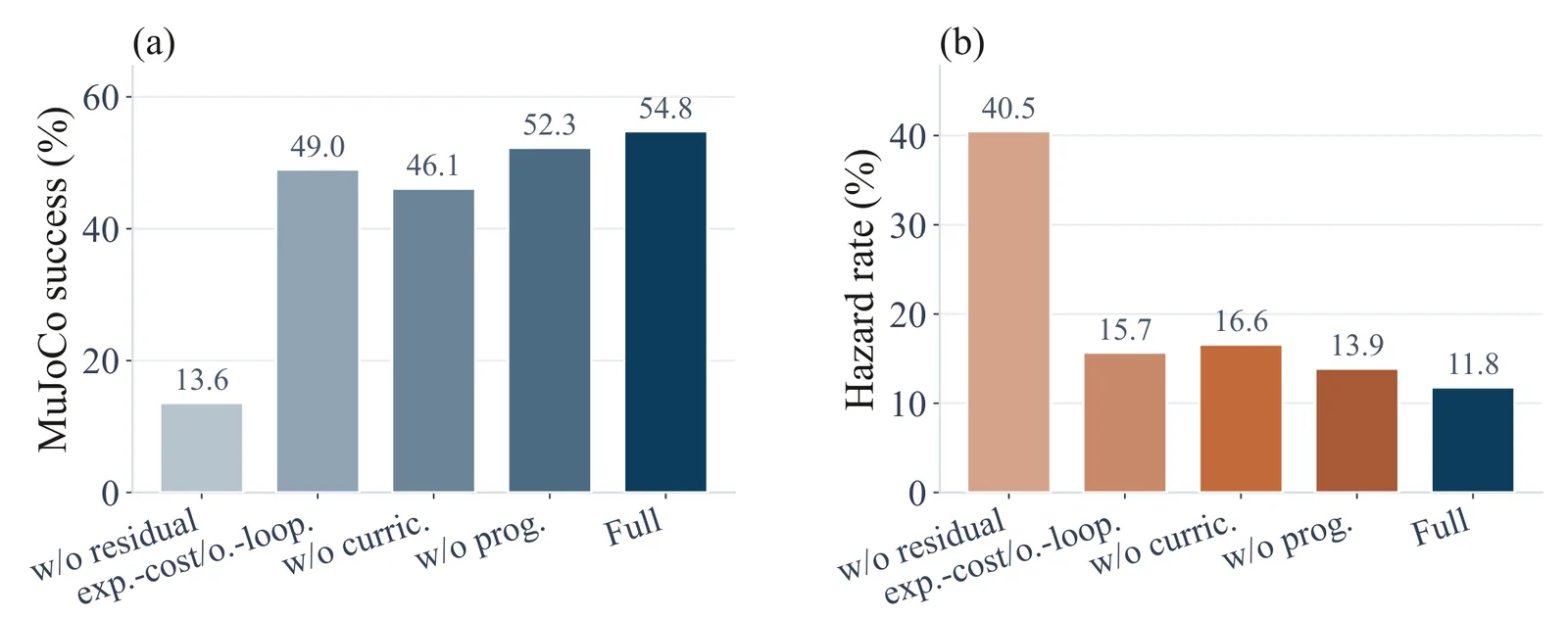
Visual summary of the MuJoCo ablation settings. (**a**) Success. (**b**) Contact/hazard rate.

**Figure 12:**
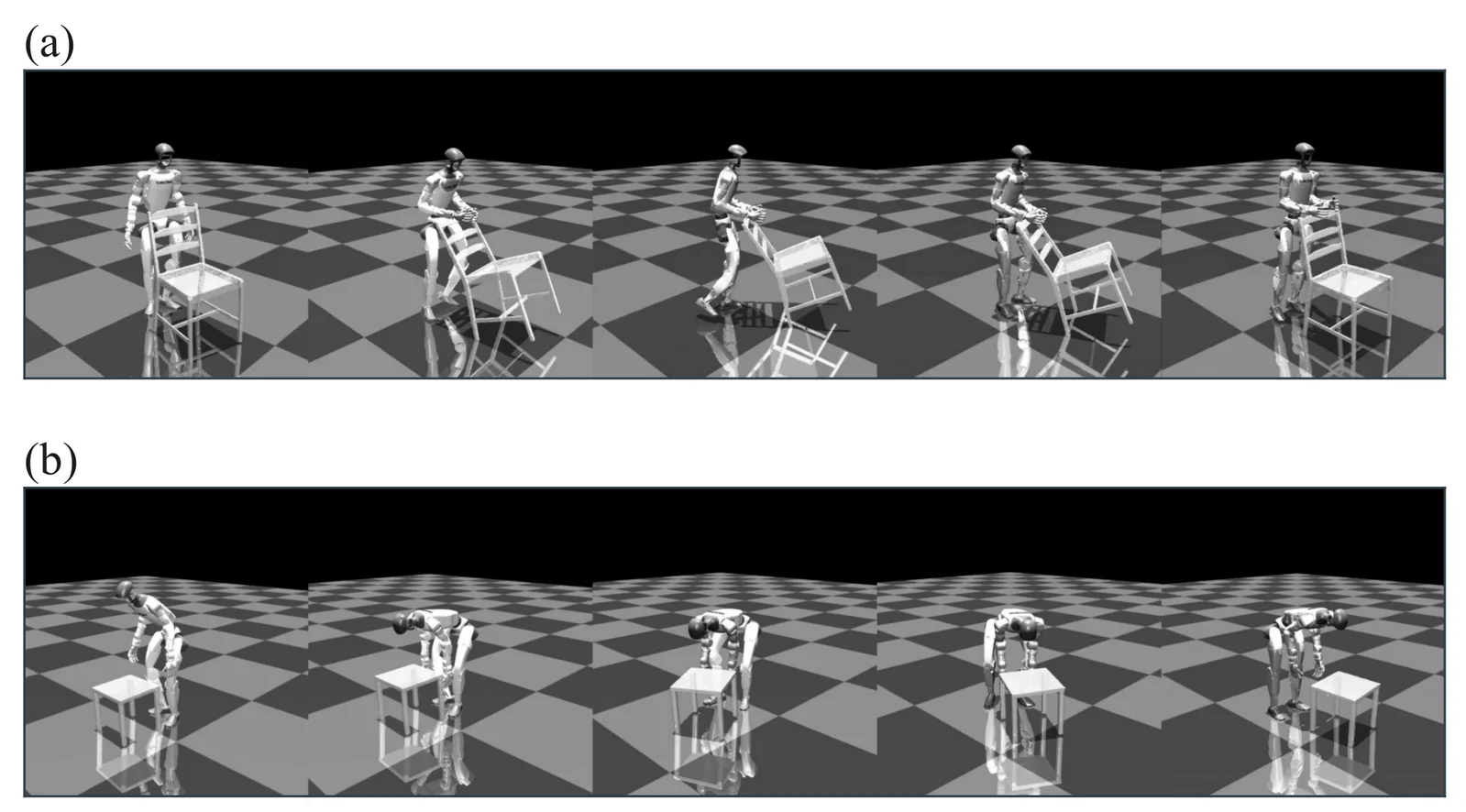
MuJoCo zero-update qualitative evidence for chair/table planar contact. (**a**) Chair-push (back contact; T1-related). (**b**) Table contact (T5 suite).

The MuJoCo ablations in Table 8, summarized in Figure 11, show 13.6% mean success without the residual policy. The expected-cost/open-loop setting removes both adaptive $\lambda_{r}$ and the deployable progression module and reaches 49.0%. Removing the curriculum gives 46.1%. Removing adaptive progression but retaining quantile-based hazard weighting gives 52.3%. The full setting reports 54.8% mean success, 11.8% hazard, and $E_{o}=0.17$ m. Figure 12 shows the corresponding planar chair/table contact behavior in zero-update MuJoCo rollouts.

**Figure 13:**
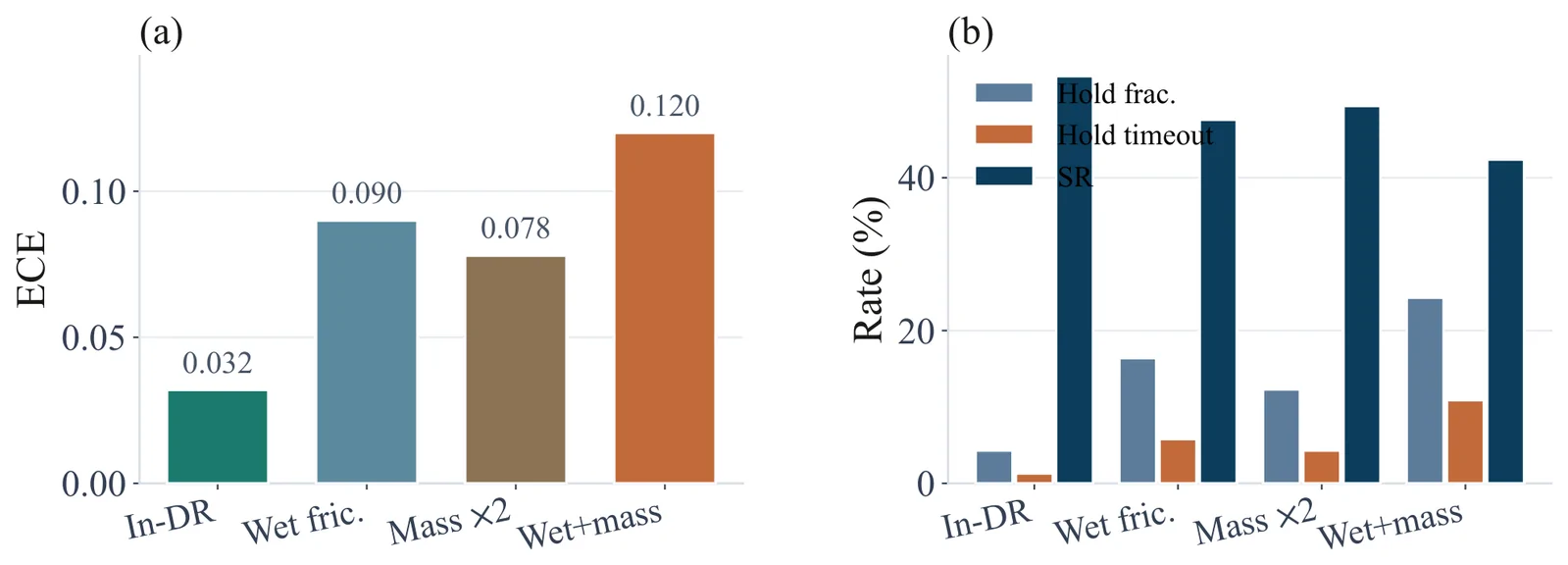
Beyond-randomization diagnostics for the deployable hazard estimator. (**a**) ECE. (**b**) Hold fraction, hold timeout, and success.

We also test the deployable hazard estimator under disturbances outside the training domain-randomization envelope; Table 9 and Figure 13 report calibration and progression statistics. On in-DR held-out transitions, the hybrid hazard score has ECE 0.032 against the 50-step event label and a low hold fraction. Ground friction far below the DR range (“wet”) or object mass scaled by 2× raises ECE to 0.090 and 0.078, respectively. Both conditions increase hold occupancy and phase-timeout terminations after prolonged hold. Under combined wet+2× mass stress, ECE reaches 0.120 and 10.9% of episodes remain on hold until timeout. The multi-component ablations are not one-factor isolations.

### 5.4. Added-Payload and Risk-Configuration Tests

**Figure 14:**
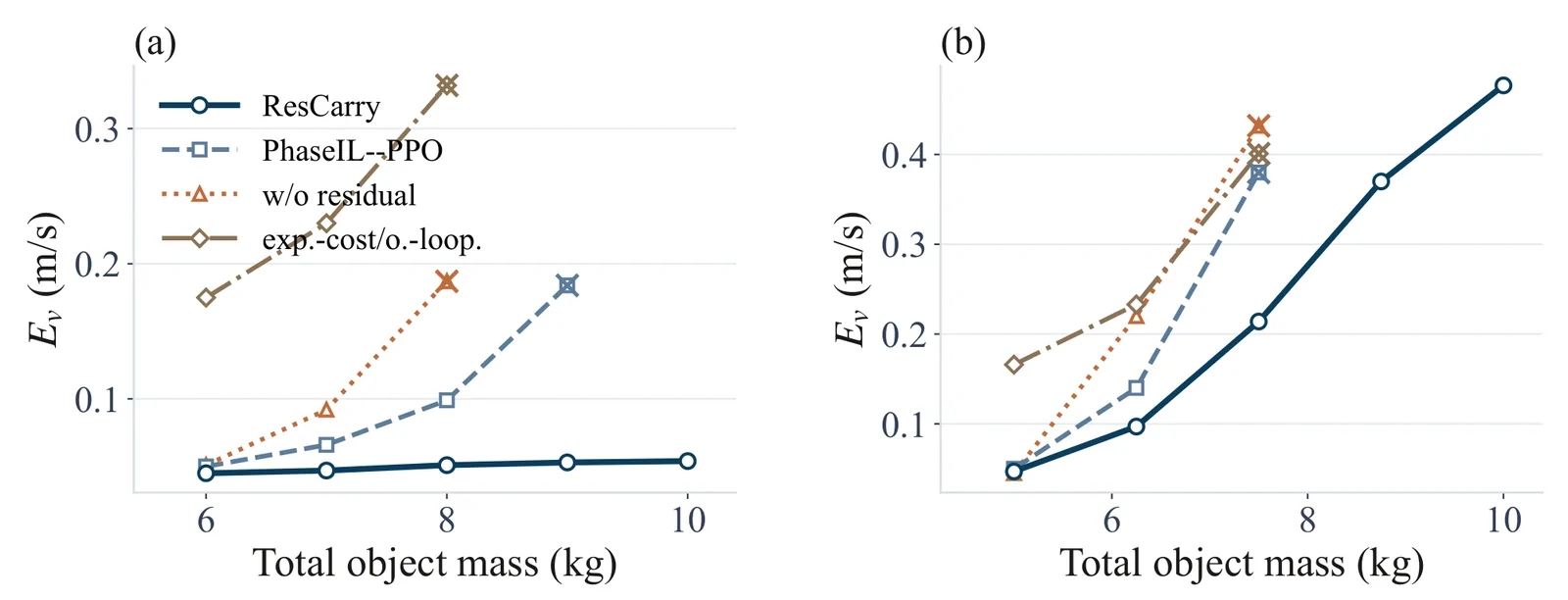
Total object mass stress on planar speed tracking $E_{v}$ (Isaac Lab; same settings as Table 10 and Table 11). (**a**) Chair-carry. (**b**) Suitcase-push. Curves stop at the last total mass that met the evaluation success criterion.

**Figure 15:**
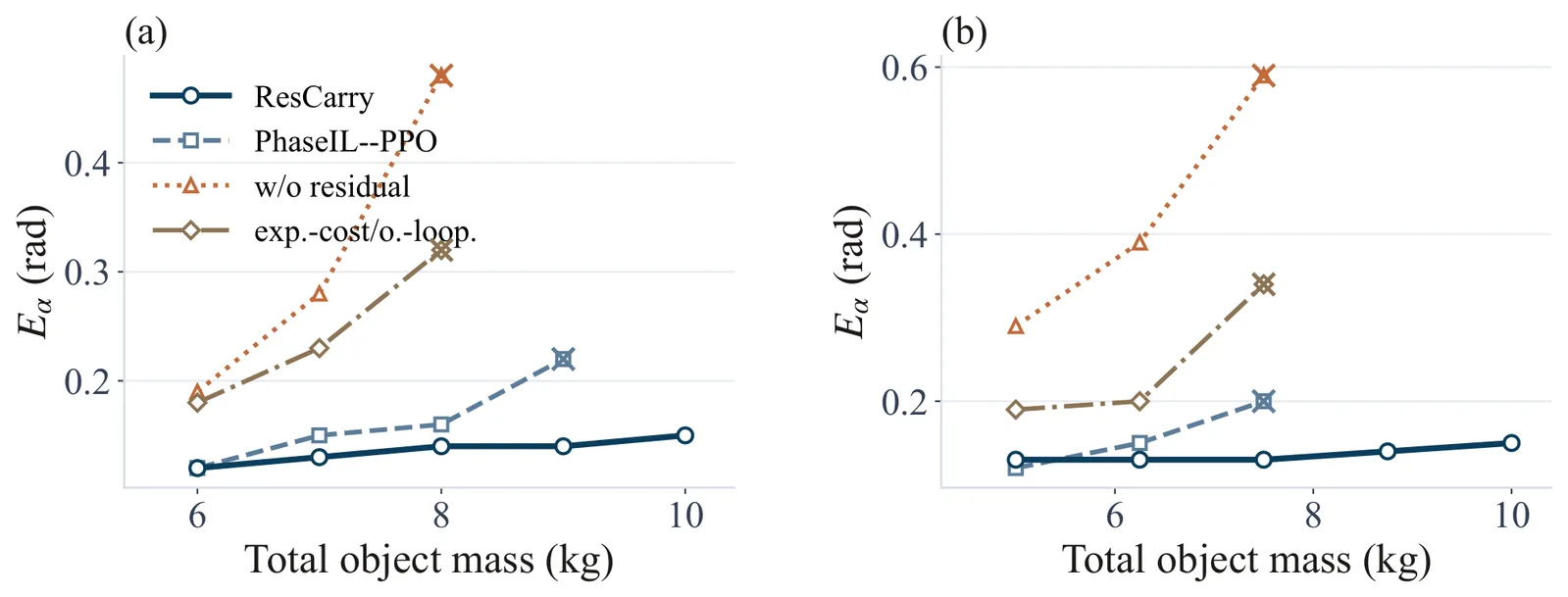
Total object mass stress on torso pitch tracking $E_{\alpha }$ (Isaac Lab; same settings as Table 10 and Table 11). (**a**) Chair-carry. (**b**) Suitcase-push.

**Figure 16:**
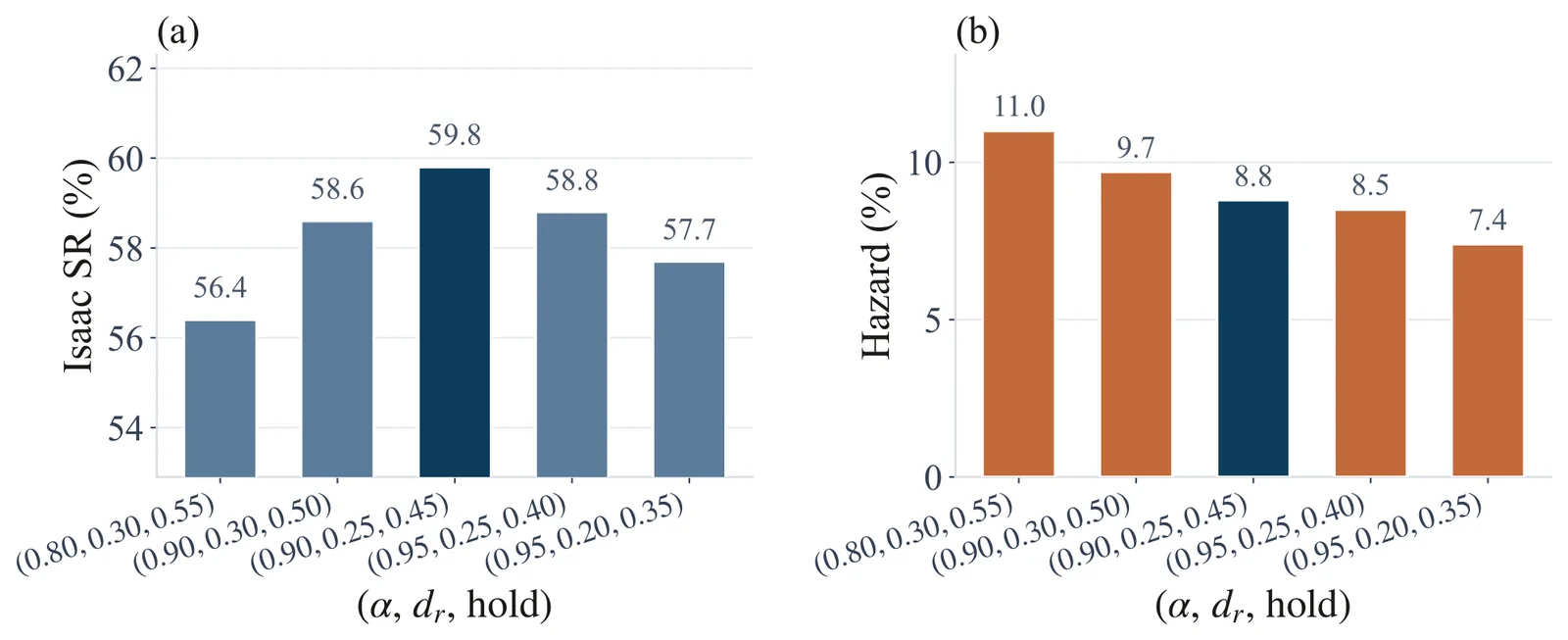
Comparison of five jointly selected risk/progression configurations; the deployed setting is highlighted. (**a**) Isaac success. (**b**) Hazard rate.

We stress-test heavier loads by fixing a representative nominal object mass per skill (Chair-carry: 6.0 kg; Suitcase-push: 5.0 kg; all tested total masses are at most 10.0 kg) and adding extra payload on top; Table 10 and Table 11 and Figure 14 and Figure 15 report Isaac Lab command-tracking errors at each total object mass. Metrics follow the residual command channels: torso pitch $E_{\alpha }$ (rad), CoM height $E_{h}$ (m), planar base speed $E_{v}$ (m/s), and yaw $E_{\psi }$ (rad). The tables report tracking errors for successful episodes. “Fail” denotes that the evaluation success criterion was not met at that total-mass setting. Five jointly selected operating configurations are compared in Table 12 and Figure 16 using the $K_{\alpha }$ atoms in (13). Because α, $d_{r}$, and the hold threshold change together, the comparison is not a one-factor sensitivity analysis. The deployed configuration gives 59.8% mean success and 8.8% mean hazard in this comparison.

## 6. Discussion

All results use simulator-provided object pose and twist; cameras and a learned state estimator are not included. Policies are trained in Isaac Lab and transferred to Unitree MuJoCo without updates under the same actor observation. During training, the quantile hazard critic receives the privileged input $o_{t}^{c}$ in (9); the deployable estimator uses only $o_{t}^{\pi }$. The beyond-DR tests in Table 9 show higher calibration error and hold occupancy outside the training envelope.

Freezing Stage I and specializing Stage II accounts for most of the gain over continued supervised prior training and the coarse-reference residual (Table 4; Figure 4 and Figure 5). Zero-update Isaac → MuJoCo transfer lowers ResCarry mean success by 4.9 points and shifts failures toward grasp loss and timeout (Table 7). No physical G1 trials are reported. Object pose and twist are idealized simulator outputs with bounded noise and latency, so real estimation errors and dropouts are not modeled. Hazard-weighted updates are a penalty schedule, not a certified safety guarantee. Future work should couple the residual controller with visual or proprioceptive object tracking and test the full system on physical Unitree G1 hardware.

Several future directions follow directly from these limitations. First, the simulator-provided object-state should be replaced by an estimator that fuses vision, proprioception, contact, and inertial cues, then tested under occlusion and intermittent contact. Second, the phase-conditioned command prior could be extended from the present per-task token generator to a graph-structured spatiotemporal generator that models contacts among robot links, object surfaces, and support regions. Recent work on dynamic spatiotemporal graph generative adversarial networks and adaptive dynamic scenario clustering shows how nonlinear spatiotemporal dependence and correlation preservation can be modeled in uncertain dynamical systems [36,37]; analogous ideas may help generate more diverse contact-rich humanoid carrying scenarios, although they are not part of the current implementation. Third, hardware deployment should study estimator delay, dropped object-state updates, actuator heating, and recovery after partial grasp loss. Fourth, the hazard-weighting rule should be compared with certified constrained-RL or model-predictive safety filters if formal safety guarantees are required.

## 7. Conclusions

We presented a two-stage whole-body carrying formulation: a fixed phase-conditioned object-centric command prior supplies the nominal reference, and a residual policy learns bounded corrections from proprioceptive, contact, and object-state observations. A quantile upper-tail score adapts the expected-cost PPO penalty, and a deployable hazard score estimator regulates command progression. Across the six-task simulation study, ResCarry reaches 59.7% mean success in Isaac Lab and 54.8% in zero-update MuJoCo, above the matched baselines. Future work will add object-state estimation and evaluate the system on physical hardware.

## Figures and Tables

**Table 1:** Network architecture summary. MLPs use orthogonal initialization and layer normalization after hidden layers; the actor uses a tanh output.

Module	Input/Output	Architecture	Role
Phase tokenizer	chunk/latent	3 convs, VQ 128×64	Stage I compression
Causal transformer	task/history/token	8 layers, 256 width, 8 heads	Stage I generation
Residual actor $\pi_{\theta }$	137-D/29-D	MLP 512–256–128	Deployable policy
Reward critic	critic obs./value	MLP 512–256–128	Reward GAE
Quantile hazard critic	critic obs./32 atoms	MLP 512–256–128	Upper-tail score
Hazard estimator $\rho_{\chi }$	actor window/score	Temporal conv, MLP 256–128	Progression

**Table 2:** Six contact-specified furniture tasks. Mass and travel ranges are randomized at episode start; contact mode is fixed by the task identifier.

ID	Task	Contact	Mass (kg)	Dist. (m)	Δψ
T1	Chair-drag	btp: Back-top press	2.5–5.0	1.5–3.0	$30^{\circ }$–$90^{\circ }$
T2	Chair-carry	sh: Seat hold	2.0–4.5	1.8–3.5	$35^{\circ }$–$100^{\circ }$
T3	Suitcase-push	sp: Side push	3.0–6.5	2.0–4.0	$40^{\circ }$–$110^{\circ }$
T4	Storage-push	sp: Side push	2.5–6.0	2.0–3.8	$35^{\circ }$–$100^{\circ }$
T5	Table-pull	uep: Under-edge	4.5–10.0	1.2–2.5	$25^{\circ }$–$80^{\circ }$
T6	Bin-carry	bh: Bilateral hug	1.5–3.5	1.8–3.5	$40^{\circ }$–$120^{\circ }$

**Table 3:** Domain randomization during residual training (paired columns for a compact wide layout).

Quantity	Range	Quantity	Range
Object mass scale	[0.65,1.40]	Object CoM offset (each axis)	[−0.05,0.05] m
Object inertia scale	[0.70,1.35]	Ground friction	[0.55,1.20]
Hand–object friction	[0.45,1.15]	Motor-strength scale	[0.85,1.15]
Joint damping scale	[0.75,1.30]	PD stiffness scale	[0.90,1.10]
Proprioceptive latency	0–40 ms	Object-state latency	0–60 ms
Command latency	0–40 ms	Joint-position noise	σ=0.004 rad
Joint-velocity noise	σ=0.05 rad/s	Object-position noise	σ=0.008 m
External base impulse	0–55 N s	External object impulse	0–45 N s

**Table 4:** Primary comparison on six furniture tasks (Isaac Lab; five-seed means). Task columns: CD = Chair-drag, CC = Chair-carry, SuP = Suitcase-push, StP = Storage-push, TP = Table-pull, BC = Bin-carry (SR %). Mean SR and tracking errors $E_{o}$/$E_{m}$ (m), $E_{j}$ (rad). Late-training success plateaus match Figure 5.

Method	CD	CC	SuP	StP	TP	BC	Mean	$E_{o}$	$E_{m}$	$E_{j}$
Base prior	16	15	20	16	14	18	16.5	0.45	2.82	2.79
Coarse-reference residual	33	34	32	30	30	30	31.5	0.33	2.10	2.25
Cont. supervised prior	42	44	42	42	37	43	41.7	0.26	1.46	1.93
PhaseIL–PPO	50	59	54	52	45	56	52.7	0.18	0.95	1.57
ResCarry	55	66	58	61	53	65	59.7	0.14	0.50	1.35

**Table 5:** Statistical summary for the primary Isaac Lab comparison. Confidence intervals use the pooled 1200 binary evaluation episodes per method (40 episodes × five seeds × six tasks). The *z* and *p* columns compare each method with ResCarry by a two-proportion test; ResCarry is the reference row.

Method	Mean SR (%)	95% CI (%)	*z* vs. ResCarry	*p* vs. ResCarry
Base prior	16.5	14.5–18.7	24.33	<$10^{-12}$
Coarse-reference residual	31.5	28.9–34.2	14.46	<$10^{-12}$
Cont. supervised prior	41.7	38.9–44.5	8.97	<$10^{-12}$
PhaseIL–PPO	52.7	49.9–55.5	3.46	$5.4\;\times \;10^{-4}$
ResCarry	59.7	56.9–62.4	–	–

**Table 6:** Unitree MuJoCo zero-update success (%) on the six furniture tasks (task abbreviations as in Table 4; Isaac per-task SR is reported there). Overall is the arithmetic mean of the six task-level values before final one-decimal rounding.

Method	CD	CC	SuP	StP	TP	BC	Overall
Coarse-reference residual	25.0	25.5	25.0	22.0	22.0	22.5	23.7
Cont. supervised prior	34.5	36.0	34.5	35.0	30.0	35.5	34.3
PhaseIL–PPO	44.0	52.5	48.0	46.5	40.5	49.5	46.9
ResCarry	50.5	61.0	54.5	55.5	48.5	59.0	54.8

**Table 7:** Failure taxonomy for full ResCarry (percent of evaluation episodes; categories need not sum to 100%).

Engine	Fall	Drop	Grasp Loss	Collision	Timeout	Placement
Isaac Lab	5.5	7.7	7.0	5.1	7.1	7.9
Unitree MuJoCo	6.3	9.0	7.3	6.4	8.4	7.8

**Table 8:** ResCarry ablations on MuJoCo (six-task mean; wide layout).

Metric	w/o Residual	Expected-Cost/Open-Loop	w/o Curric.	w/o Progression	Full
SR (%)	13.6	49.0	46.1	52.3	54.8
Hazard (%)	40.5	15.7	16.6	13.9	11.8
$E_{o}$ (m)	0.47	0.21	0.21	0.19	0.17

**Table 9:** Hazard score calibration diagnostic and progression behavior under beyond-DR disturbances (MuJoCo, six-task mean). ECE uses logit temperature scaling on held-out transitions (15 bins); hold frac. = share of steps with $\rho_{\chi }\geq \rho_{\mathrm{hld}}$; hold timeout = prolonged conservative hold until phase timeout.

Condition	ECE	Hold Frac. (%)	Hold Timeout (%)	SR (%)
In-DR (held-out)	0.032	4.3	1.3	53.3
OOD: wet friction (below DR)	0.090	16.4	5.8	47.6
OOD: object mass ×2	0.078	12.3	4.3	49.4
OOD: wet + mass ×2	0.120	24.3	10.9	42.4

**Table 10:** Total object mass stress on Chair-carry (lift/carry; Isaac Lab; nominal 6.0 kg chair plus added payload; totals ≤10.0 kg). Lower is better; Fail marks episode collapse under the evaluation protocol.

Total Mass (kg)	Method	$E_{\alpha }$	$E_{h}$	$E_{v}$	$E_{\psi }$
6	ResCarry	0.12	0.013	0.045	0.14
	PhaseIL–PPO	0.12	0.011	0.050	0.13
	w/o residual	0.19	0.050	0.051	0.41
	expected-cost/open-loop	0.18	0.022	0.175	0.11
7	ResCarry	0.13	0.016	0.047	0.14
	PhaseIL–PPO	0.15	0.021	0.066	0.15
	w/o residual	0.28	0.082	0.092	0.53
	expected-cost/open-loop	0.23	0.040	0.230	0.13
8	ResCarry	0.14	0.016	0.051	0.14
	PhaseIL–PPO	0.16	0.032	0.099	0.16
	w/o residual	0.48	0.133	0.187	0.63
	expected-cost/open-loop	0.32	0.073	0.332	0.21
9	ResCarry	0.14	0.016	0.053	0.15
	PhaseIL–PPO	0.22	0.064	0.184	0.20
	w/o residual	Fail	Fail	Fail	Fail
	expected-cost/open-loop	Fail	Fail	Fail	Fail
10	ResCarry	0.15	0.016	0.054	0.15
	PhaseIL–PPO	Fail	Fail	Fail	Fail
	w/o residual	Fail	Fail	Fail	Fail
	expected-cost/open-loop	Fail	Fail	Fail	Fail

**Table 11:** Total object mass stress on Suitcase-push (side push; Isaac Lab; nominal 5.0 kg suitcase plus added payload; totals ≤10.0 kg). Lower is better; Fail marks episode collapse under the evaluation protocol.

Total Mass (kg)	Method	$E_{\alpha }$	$E_{h}$	$E_{v}$	$E_{\psi }$
5	ResCarry	0.13	0.013	0.047	0.13
	PhaseIL–PPO	0.12	0.016	0.050	0.13
	w/o residual	0.29	0.045	0.045	0.42
	expected-cost/open-loop	0.19	0.025	0.166	0.12
6.25	ResCarry	0.13	0.015	0.097	0.13
	PhaseIL–PPO	0.15	0.019	0.140	0.16
	w/o residual	0.39	0.074	0.220	0.56
	expected-cost/open-loop	0.20	0.040	0.233	0.14
7.5	ResCarry	0.13	0.017	0.214	0.14
	PhaseIL–PPO	0.20	0.050	0.380	0.23
	w/o residual	0.59	0.176	0.432	0.76
	expected-cost/open-loop	0.34	0.070	0.401	0.21
8.75	ResCarry	0.14	0.015	0.370	0.15
	PhaseIL–PPO	Fail	Fail	Fail	Fail
	w/o residual	Fail	Fail	Fail	Fail
	expected-cost/open-loop	Fail	Fail	Fail	Fail
10	ResCarry	0.15	0.017	0.477	0.17
	PhaseIL–PPO	Fail	Fail	Fail	Fail
	w/o residual	Fail	Fail	Fail	Fail
	expected-cost/open-loop	Fail	Fail	Fail	Fail

**Table 12:** Comparison of jointly selected upper-tail, reference-value, and hold-threshold configurations (Isaac Lab, six-task mean).

Upper-Tail Level α	Reference Value $d_{r}$	Hold Threshold	Isaac SR (%)	Hazard (%)	Time (s)
0.80	0.30	0.55	56.4	11.0	19.4
0.90	0.30	0.50	58.6	9.7	19.6
0.90	0.25	0.45	59.8	8.8	20.9
0.95	0.25	0.40	58.8	8.5	21.7
0.95	0.20	0.35	57.7	7.4	23.8

## Data Availability

The benchmark configurations, evaluation protocols, and aggregate results are reported in this article. No separate supplementary data or code files accompany this article.

## Abbreviations

The following abbreviations are used in this manuscript:

ResCarry	Residual learning for humanoid object carrying
HDMI	Human-video-based interactive humanoid whole-body control method
HuMI	Humanoid Manipulation Interface
OMOMO	Object Motion guided human Motion synthesis
SEEC	Stable End-Effector Control
FALCON	Force-adaptive humanoid loco-manipulation framework
TRILL	Teleoperation-based humanoid loco-manipulation learning framework
HALO	Heterogeneous-agent Lyapunov policy optimization
CVaR	Conditional Value-at-Risk
VaR	Value-at-Risk
PPO	Proximal Policy Optimization
IL	Imitation Learning
RL	Reinforcement Learning
HOI	Human–Object Interaction
DoF	Degrees of Freedom
VQ	Vector Quantization
ECE	Expected Calibration Error
IMU	Inertial Measurement Unit
IK	Inverse Kinematics
PD	Proportional–Derivative
GAE	Generalized Advantage Estimation
DR	Domain Randomization
CMDP	Constrained Markov Decision Process
RMS	Root-Mean-Square

## Appendix A. Empirical CVaR Score and Dual-Update Interpretation

For a scalar cost return *C*, the standard conditional value at risk is

(A1) $${\mathrm{CVaR}}_{\alpha }\left(C\right)=\min_{\nu }\left\{\nu +\frac{1}{1-\alpha }E\left[{\left(C-\nu \right)}_{+}\right]\right\}=\frac{1}{1-\alpha }\int_{\alpha }^{1}{\mathrm{VaR}}_{u}\left(C\right)\;du,$$

i.e., the average of the upper (1−α) tail of the return, where ${\mathrm{VaR}}_{u}$ denotes the value-at-risk (VaR) at level *u*. The quantile critic outputs $\left\{z_{m}\left(o_{t}^{c}\right)\right\}$ approximate ${\mathrm{VaR}}_{\tau_{m}}\left(Z_{t}^{c}\right)$ at levels $\tau_{m}=\left(m-0.5\right)/M$. Their tail average in (13) is the standard finite-atom estimator of this integral; it converges to population ${\mathrm{CVaR}}_{\alpha }$ as M→∞ and is a quantile-discretization approximation at finite *M*. The actor uses a clipped expected-cost advantage, and the empirical CVaR score drives the projected dual-ascent update of $\lambda_{r}$ in (18). The progression law in (20) reduces or pauses command progress as the estimated hazard score rises. Its effect is evaluated through the open-loop-progression comparison and the reported timeout statistics.

## Appendix B. Command Prior and Phase Automaton

Demonstrations use $N_{\mathrm{omo}}\;=\;1800$ OMOMO-retargeted G1 clips (300 per task) and $N_{\mathrm{tch}}\;=\;600$ privileged A*/minimum-jerk teacher trajectories (100 per task) that place task-specific contact anchors (back-top, seat, side, under-edge, hug); both sources are used in the reported experiments (N=2400 total). Table A1 lists the contact-phase automaton shared by labeling and execution. Commands are normalized to [−1,1] before VQ training (base $v_{x,y}$, yaw rate, CoM height, torso pitch, wrist Δp/Δr, apertures). Phase encoders use three temporal convolutions to 64-D latents; the causal transformer (width 256, eight heads, context 32) emits phase-specific tokens with H=16 chunks blended by a four-step half-cosine window.

**Table A1:** Contact-phase automaton for demonstration labeling and learned execution.

Phase	Entry	Exit	Timeout
app	Episode start	Wrist err. <50 mm, base speed <0.10 m/s for 0.15 s	8.0 s
sec	app exit	Grippers closed, contacts active 0.20 s	2.0 s
lift	sec exit	Object rise >0.12 m, tilt $<10^{\circ }$ for 0.20 s	2.5 s
trn	lift exit	At target, yaw err. $<15^{\circ }$ for 0.20 s	25.0 s
plc	trn exit	Support 0.30 s, grippers open	4.0 s

## Appendix C. Residual RL Hyperparameters and Actor Surrogate

The nominal resolver consists of a frozen lower-body velocity tracker and damped arm IK with $\lambda_{\mathrm{ik}}\;=\;0.04$. The deployable observation ($d_{\pi }\;=\;137$) stacks joint error/velocity (29+29), base IMU/gravity/velocity (3+3+3), command (19), object pose as position+axis-angle (6), object twist (6), contacts (4), previous residual (29), phase one-hot (5), and chunk progress (1); object pose is not encoded as a quaternion or rotation matrix. Hazard detection uses the following thresholds: fall for base height <0.55 m or tilt $>50^{\circ }$; drop for an unsupported object with no gripper contact; grasp loss for one-sided contact loss > 0.10 s; collision for unintended contact excluding feet and grasp; slip for tangential foot speed > 0.12 m.s^−1^ during stance; and joint-limit proximity within $2^{\circ }$ of a hard limit while moving toward it. Episodes terminate on fall, drop, high-impulse collision, or hard joint limits, while one-sided grasp loss triggers hazard with a 0.20 s recovery window. Reward terms specialize $r_{\mathrm{bas}}$, $r_{\mathrm{wri}}$, $r_{\mathrm{grp}}$, $r_{\mathrm{up}}$, and phase-dependent $r_{\mathrm{obj}}$ as in Section 3.4; numeric weights are listed in Section 4.

For cost GAE, let $\bar{z}\left(o^{c}\right)=\frac{1}{M}\sum_{m}z_{m}\left(o^{c}\right)$ denote the mean quantile atom with stop-gradient. The TD cost residual $\delta_{t}^{c}=c_{t}+\gamma_{c}\left(1-d_{t}\right)\bar{z}\left(o_{t+1}^{c}\right)-\bar{z}\left(o_{t}^{c}\right)$ yields ${\hat{A}}_{t}^{c}$ with $\lambda_{\mathrm{gae}}=0.95$ and the same horizon handling as the reward GAE ${\hat{A}}_{t}^{r}$. The actor maximizes (17), with the upper-tail batch statistic stopped with respect to the actor; Actor Adam uses $3\;\times \;10^{-4}$, and PhaseIL–PPO uses fixed $\lambda_{r}\;=\;0.12$ without quantile-based adaptation. Temperature scaling for ECE is applied to pre-sigmoid logits on N=12,800 held-out pairs using 15 equal-width bins over [0,1], while $Z_{\max}^{c}$ is an empirical clip near the 99th percentile of training critic returns (Section 4). The actor and critic use MLPs with orthogonal initialization; the hazard critic is 512–256–128 with *M* quantile heads and Polyak coefficient 0.995, and the hazard estimator uses a temporal convolution followed by an MLP. PD gains are (100,2.5) for hip/knee, (40,1.2) for ankles, (45,1.2) for arms, and (25,0.8) for wrists. The joint configuration comparison for $\left(\alpha ,d_{r},\rho_{\mathrm{hld}}\right)$ is reported in Table 12.
